# A systems medicine approach for finding target proteins affecting treatment outcomes in patients with non-Hodgkin lymphoma

**DOI:** 10.1371/journal.pone.0183969

**Published:** 2017-09-11

**Authors:** Faezeh Ajorloo, Mohammad Vaezi, Alireza Saadat, Seyed Reza Safaee, Behrouz Gharib, Mostafa Ghanei, Seyed Davar Siadat, Farzam Vaziri, Abolfazl Fateh, Mehrdad Pazhouhandeh, Behrouz Vaziri, Reza Moazemi, Fereidoun Mahboudi, Fatemeh Rahimi Jamnani

**Affiliations:** 1 Department of Biology, Faculty of Science, Islamic Azad University, East Tehran Branch, Tehran, Iran; 2 Human Antibody Lab, Innovation Center, Pasteur Institute of Iran, Tehran, Iran; 3 Hematology-Oncology and Stem Cell Transplantation Research Center, Tehran University of Medical Sciences, Tehran, Iran; 4 Department of Hematology & Oncology, Baqiyatallah University of Medical Sciences, Tehran, Iran; 5 Hematology and Oncology Research Center, Imam Khomeini Hospital Complex, Tehran University of Medical Sciences, Tehran, Iran; 6 Department of Internal Medicine (Hematology and Oncology), Qom University of Medical Sciences, Qom, Iran; 7 Chemical Injuries Research Center, Baqiyatallah University of Medical Sciences, Tehran, Iran; 8 Microbiology Research Center, Department of Mycobacteriology and Pulmonary Research Pasteur Institute of Iran, Tehran, Iran; 9 Biotechnology Research Center, Pasteur Institute of Iran, Tehran, Iran; CCAC, UNITED STATES

## Abstract

Autoantibody profiling with a systems medicine approach can help identify critical dysregulated signaling pathways (SPs) in cancers. In this way, immunoglobulins G (IgG) purified from the serum samples of 92 healthy controls, 10 pre-treated (PR) non-Hodgkin lymphoma (NHL) patients, and 20 NHL patients who underwent chemotherapy (PS) were screened with a phage-displayed random peptide library. Protein-protein interaction networks of the PR and PS groups were analyzed and visualized by Gephi. The results indicated AXIN2, SENP2, TOP2A, FZD6, NLK, HDAC2, HDAC1, and EHMT2, in addition to CAMK2A, PLCG1, PLCG2, GRM5, GRIN2B, GRIN2D, CACNA2D3, and SPTAN1 as hubs in 11 and 7 modules of PR and PS networks, respectively. PR- and PS-specific hubs were evaluated in the Kyoto Encyclopedia of Genes and Genomes (KEGG) and Reactome databases. The PR-specific hubs were involved in Wnt SP, signaling by Notch1 in cancer, telomere maintenance, and transcriptional misregulation. In contrast, glutamate receptor SP, Fc receptor-related pathways, growth factors-related SPs, and Wnt SP were statistically significant enriched pathways, based on the pathway analysis of PS hubs. The results revealed that the most PR-specific proteins were associated with events involved in tumor development, while chemotherapy in the PS group was associated with side effects of drugs and/or cancer recurrence. As the findings demonstrated, PR- and PS-specific proteins in this study can be promising therapeutic targets in future studies.

## Introduction

Non-Hodgkin lymphoma (NHL) constitutes a highly heterogeneous group of lymphoproliferative malignancies, arising from both B and T lymphocytes, as well as natural killer cells [1, 2]. According to statistics, 70 800 new cases of NHL were identified in the USA in 2014, 96 788 new cases were diagnosed in Europe in 2015, and different occurrences have been reported in various countries. Overall, NHL remains among the top 10 most frequent cancers in the world [3].

In many NHL subtypes, timely diagnosis can result in effective and often curative treatment [4]. Today, rituximab, cyclophosphamide, doxorubicin, vincristine, and prednisone (R-CHOP) are the treatment of choice for different subtypes of NHL. However, many patients with relapsed or primary refractory NHL cannot be treated by standard therapy, and generally show poor prognosis [3, 5].

Considering NHL relapse and chemoresistance as major complications of treatment, there is an urgent need for developing novel drugs which target tumor antigens in the involved pathways. Nevertheless, only a few effective targets, such as B-lymphocyte antigen, CD20, have been introduced so far. CD20 has shown major therapeutic effects given its involvement in the pathogenicity of a wide range of diseases including NHL [6].

The generation of autoantibodies (AAbs) targeting tumor antigens has provided opportunities for using the immune system as a source for finding therapeutic targets. AAbs are produced in either early stages of cancer or during treatment due to different alterations, such as mutations, translocation, and posttranslation modification (PTM), resulting in the recognition of self-antigens as non-self antigens [7, 8]. Following the production of various B lymphocytes against autoantigens, some differentiate into memory cells and some into plasma cells secreting AAbs [9]. Overall, AAbs are stable and frequent proteins with a long half-life, unlike their corresponding autoantigens [9]. Therefore, assessment of AAb repertoires in cancer patients may help identify biomarkers and explain the role of important pathways in disease development. Moreover, it can help evaluate immune responses to determine the efficacy of current and novel therapeutic agents and assess their predictive role in disease recurrences or favorable clinical outcomes [9, 10].

Several studies have evaluated the AAb repertoire of NHL patients and reported anti-nuclear antibodies as dominant AAbs generated by B cells against self-antigens [11, 12]. However, in the present study, we aimed to investigate the AAb profiles of NHL patients before chemotherapy (PR) and after chemotherapy (PS), using a phage-displayed peptide library to find proteins which may be involved in tumor development, chemotherapy resistance, and cancer relapse, and are common among different NHL subtypes.

We selected a large population of healthy subjects to do a subtract panning. Two panels of PR- and PS-specific peptides were achieved by panning on the purified IgG from the sera of NHL patients. Proteins predicted by experimentally detected peptides were evaluated using protein-protein interaction databases. Furthermore, we followed-up the PS group after two years to determine if the data matched events which were predicted according to the identified pathways. Conclusively, the results demonstrated that the proteins found in this study were involved in the pathogenesis of NHL and side effects of chemotherapy.

## Materials and methods

### Sample collection

Patients, aged 18–77 years, who were diagnosed with stage II–IV disease or stage I disease with bulk, were selected during 2012–2014 from Shariati, Imam, and Baqiyatallah hospitals, Tehran, Iran (S1 Table). All samples were pathologically confirmed by local pathologists. The stage of lymphoma was defined by the referring physician, based on the Cotswolds modification of the Ann Arbor staging system [13]. To determine the common molecular pathogenesis among different NHL subtypes, we involved NHL subtypes according to the World Health Organization (WHO) classification using the International Lymphoma Epidemiology Consortium (InterLymph) guidelines [14]. Of note, patients with a history of other malignancies or active autoimmune disorders were excluded from the study. Finally, peripheral blood samples were collected from 10 NHL patients right before the onset of treatment (PR), as well as 20 patients who either were under treatment or had undergone chemotherapy (PS). In the PS group, R-CHOP and CHOP were the preferred treatments. In addition, granulocyte-colony stimulating factor (Filgrastim or lenograstim) was used for most patients in this group to alleviate or prevent neutropenia. Radiotherapy was applied for areas of primary bulky disease.

As control, we enrolled 127 healthy, age- and gender-matched individuals, who were randomly selected (age range, 11–80 years; mean age, 37.8 years). Healthy subjects were evaluated and excluded if had one or more of following conditions: positive rheumatoid factor, C-reactive protein higher than 6 mg/dL, erythrocyte sedimentation rate greater than 32 mm/h, complete blood count and chest X-ray abnormalities. All healthy participants were refrained from using anti-inflammatory drugs for three days before blood collection (S2 Table). The volunteers were interviewed to assess their demographic information and risk factors for autoimmune diseases and cancers (eg, history of diseases in patients and their families).

This study was done in accordance with the Helsinki Declaration and was approved by the HORCSCT Review Board and Ethics Committee in Shariati Hospital, the Ethical Committee of the Cancer Institute (Imam Khomeini Hospital), and the Ethics Committee of Baqiyatallah University of Medical Sciences. All participants provided written informed consent before enrollment.

### Preparation of sera and IgG purification

IgG antibodies from the pooled sera of healthy, PR, and PS subjects were individually purified using a Melon Gel IgG Purification Kit (Pierce, Rockford, IL, USA) according to the manufacturer's instructions. The purification accuracy was confirmed by reducing sodium dodecyl sulfate-polyacrylamide gel electrophoresis (SDS-PAGE) analysis.

### Biopanning

A random peptide library (Ph.D.-C7C Phage Display Peptide Library Kit, New England Biolabs, Beverly, MA, USA) was used to perform three successive cycles of biopanning on the purified IgG of NHL patients according to the manufacturer's instructions [15].

### Phage ELISA

To evaluate the specificity of phages to the PR and PS IgG, polyclonal and monoclonal phage ELISA assays were done according to the manufacturer's instructions (Ph.D.TM-C7C Kit). See supporting information (S1 Text) for details.

### DNA sequencing

Single-stranded DNAs of 22 phages (11 phages from PR and 11 phages from PS) were extracted and sequenced according to Ph.D.-C7C Kit instructions. The amino acid sequences were deduced with Gene Runner program version 5.0 and checked in the Biopanning Data Bank (MimoDB) (http://immunet.cn/bdb/) [16]. The selected peptides were blasted for *Homo sapiens* proteins, using the BLASTP tool and Refseq protein database [17].

### Gene Ontology and pathway enrichment analysis of predicted genes selected by literature research

**Fig 1:**
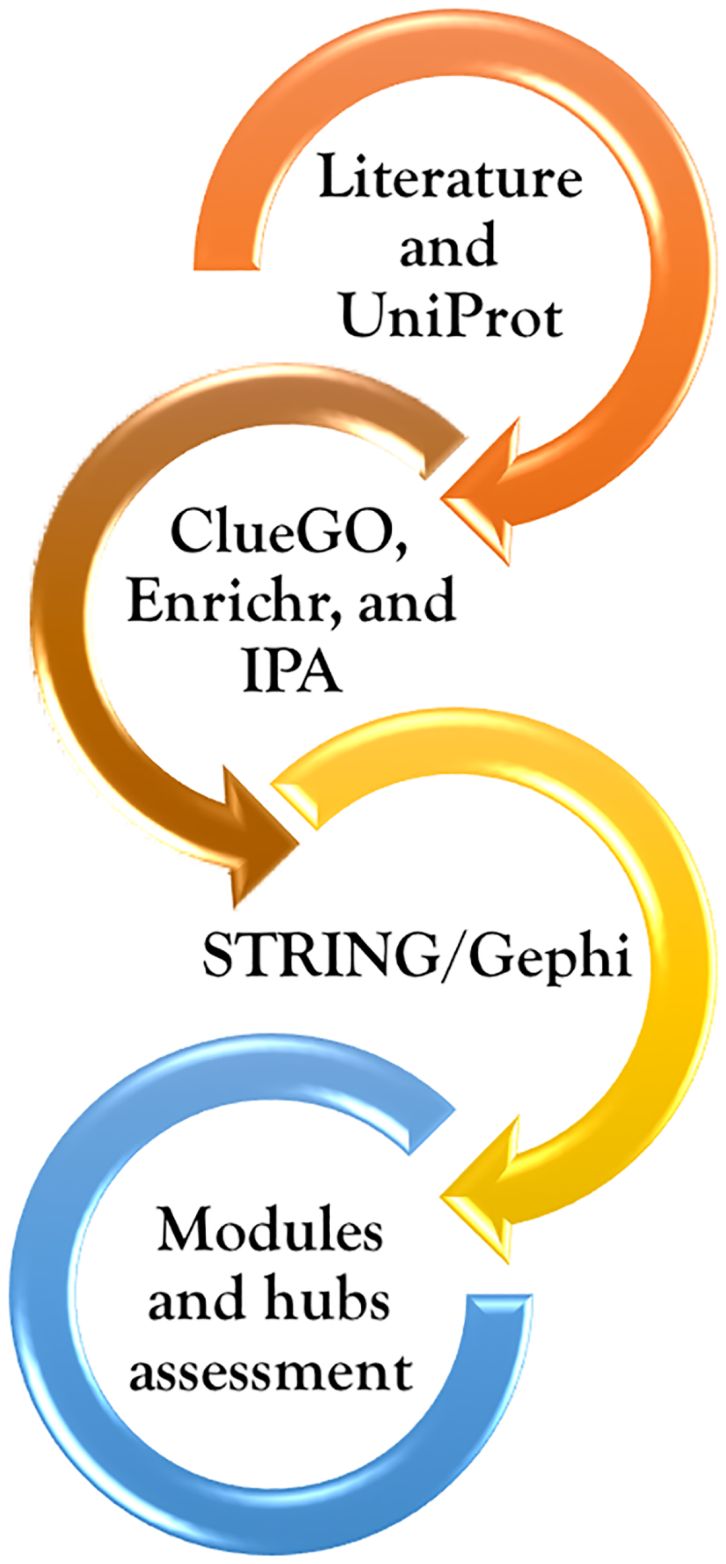
The schematic diagram of analyses in this study.

Among proteins deduced from the detected peptides, proteins associated with cancers or the immune system were extracted by searching the literature and UniProt database (Fig 1).

To identify the biological processes associated with PR and PS gene lists, and determine the correlation of overrepresented terms, Enrichr web tool (http://amp.pharm.mssm.edu/Enrichr/) and ClueGo plugin v2.3.2 [18] in Cytoscape 3.4.0 [19] were used. All results from ClueGO were based on the Ontology GO Biological Process with a Kappa Score Threshold = 0.4. To compute enriched terms and to correct *P*-value, a two-sided hypergeometric (Enrichment/Depletion) test and Benjamini—Hochberg correction were applied, respectively. Gene Ontology (GO) terms with *P* < 0.05 were considered as significant [20, 21].

To achieve enriched pathways showing the best connections among genes, pathway enrichment analysis was performed via Enrichr, based on the Kyoto Encyclopedia of Genes and Genomes (KEGG), WikiPathways, Reactome, BioCarta, Panther, and Ingenuity Pathway Analysis (IPA, QIAGEN Redwood City, www.qiagen.com/ingenuity) databases. The significance level was set at 0.05, and the minimum number of genes for each term was set at two. Additionally, ClueGO was used for verifying the pathway enrichment analysis according to the KEGG, Reactome, and WikiPathways databases. Terms selection was based on the abovementioned options.

Disease association analysis of genes was carried out using WEB-based GEne SeT AnaLysis Toolkit (WebGestalt) (http://www.webgestalt.org) and IPA. The WebGestalt database was utilized according to the parameters of a hypergeometric test for the enrichment analysis at *P* < 0.05 after BH correction [22].

### Network analysis and visualization

To precisely evaluate pathways and visualize the connection among target genes, two PR and PS gene lists were imported into STRING version 10.0 (http://string-db.org) [23]. By extracting the combined scores from STRING, as edge weights (threshold, 0.4577), different topological properties of networks, such as degree, modularity, and betweenness centrality of nodes (as representatives of proteins), were visualized and computed via Gephi 0.9.1 (http://gephi.github.io/) [23–25]. Thereby, we could identify top ranked proteins/hubs in the PR and PS networks. The GO and pathway enrichment analysis of the detected modules and pathway analysis of hubs were carried out using Enrichr (based on the KEGG and Reactome databases) to distinguish the overrepresented terms at *P* < 0.05 [26]. Additionally, PR- and PS-specific hubs were investigated through literature mining to pinpoint alterations such as expression patterns, mutations, translocations, and different modifications (e.g., methylation) which have been reported in different cancers. Moreover, their involvement in various pathways with considerable effects on tumor development, cancer progression, and chemotherapy resistance was evaluated.

### Hubs assessment in DrugBank

To find interactions between hubs and drugs and to identify relations between NHL drugs and PR- and PS-specific proteins, PR and PS hubs in addition to drugs involved in R-CHOP chemotherapy, were assessed with the DrugBank database (https://www.drugbank.ca).

### B‌‌inding of the selected hubs to the sera of PR and PS patients

SENP2 (Bioclone) and PLCG1 (ORIGENE) were assessed by ELISA as PR and PS hubs, respectively (See Supporting Information (S1 Text) for details).

### Follow-up of NHL patients who underwent chemotherapy

Patients who experienced chemotherapy were followed-up for two years and classified according to the treatment response, relapse, primary refractory, and progression under therapy [5, 27, 28]. See Supporting Information (S1 Text) for details.

### Statistical analysis

All statistical analyses were carried out using GraphPad prism. The data are presented as mean ± SD. Statistical significance was determined by a two-tailed student *t* test. *P*-value <0.05 was considered statistically significant.

## Results

### Identification of PR- and PS-specific peptides via library enrichment on NHL IgG

**Fig 2:**
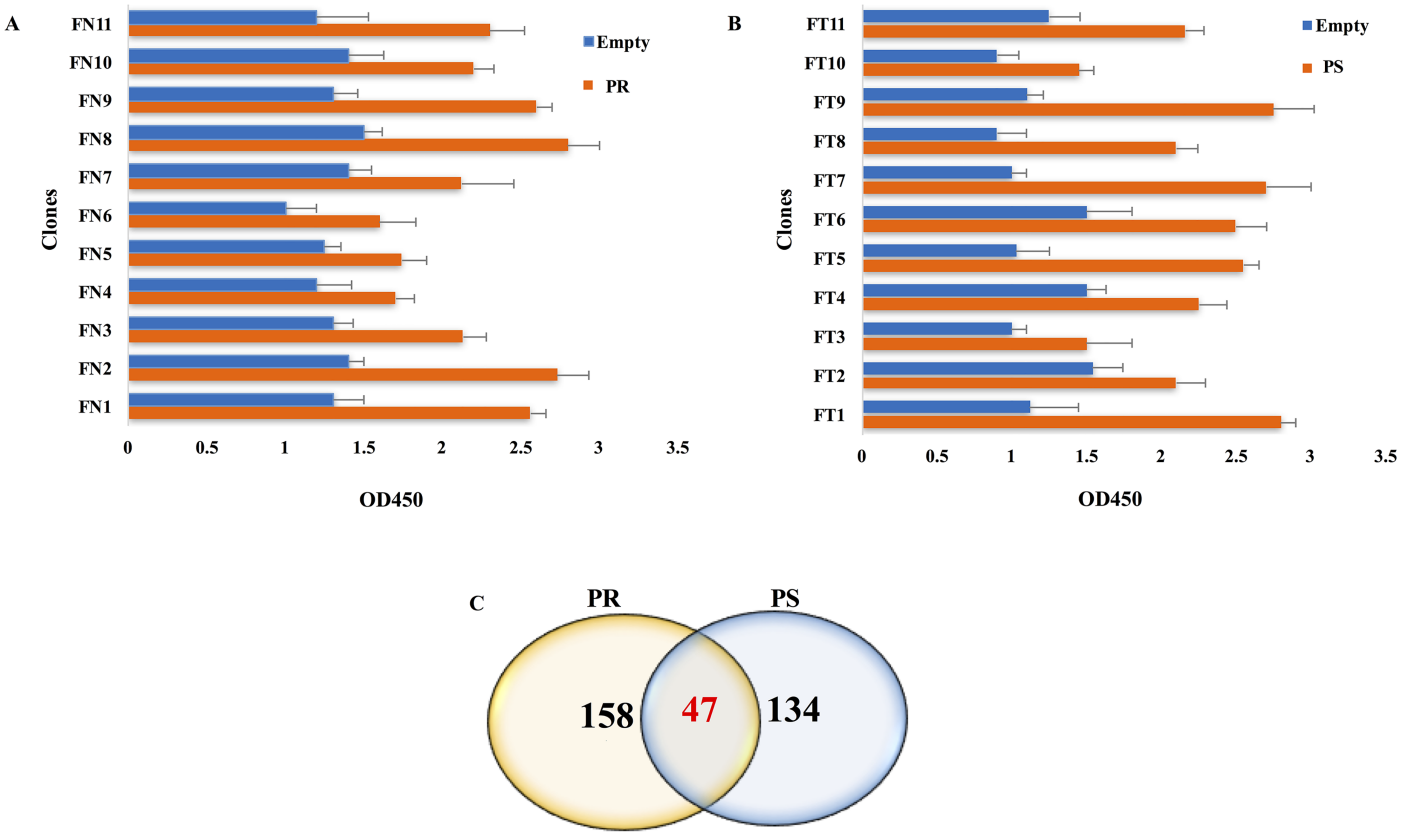
The results of monoclonal phage ELISA and Venn diagram of the predicted proteins. (A) Following the evaluation of 30 phage clones, 11 clones showed higher signal intensities in binding to the immobilized IgG of PR group (orange) compared with empty wells as the negative controls (blue). (B) 11 clones showed strong binding to the immobilized IgG of PS group (orange). (C) The Venn diagram of 158 PR, 134 PS, and 47 common proteins, which were deduced from 16 peptides via BLASTP, is presented.

To determine IgG antibodies, which are only present in the sera of NHL patients, we enrolled a large healthy population to remove AAbs which may be also found in the serum of healthy subjects and affect the results. Therefore, among 123 healthy subjects, 31 were excluded due to different abnormalities in their blood assay and chest X-ray (S2 Table). We first incubated phages (10^13^ cfu) with immobilized purified IgG from healthy controls which could entrap many unrelated phages. After incubation with purified PR and PS IgG, two pools of NHL-specific phages were obtained. During three consecutive rounds of panning, the titer of phages showed the favorite ratio of inputs to outputs consisting of enriched phages. To monitor the success of panning process, polyclonal phage ELISA was performed for PR- and PS-related inputs and outputs. Compared with the control, the greatest signals were observed in the phages of the third round of panning on the purified IgG of NHL patients. After screening 60 NHL-specific phage clones from the third round of panning (30 clones from the PR group and 30 clones from the PS group), 11 phage clones were selected for further analysis from each NHL group; they showed significant signals compared with the control on monoclonal phage ELISA (Fig 2A and 2B).

After DNA sequencing, 8 and 9 clones yielded acceptable sequences from the PR and PS groups, respectively (S3 Table). According to the MimoDB database, all sequences were target-related peptides. As shown in S3 Table, the amino acid sequences of clone FN7 of the PR group and clones FT2 and FT4 in the PS group were identical.

According to the default parameters defined in BLASTP, 1100 proteins with scores above 18.5 were identified, among which 205 PR proteins and 181 PS proteins were finally selected, based on the primary evaluations via mining the literature and UniProt database to determine if they were related to events involved in cancer or autoimmunity (Fig 2C). Accordingly, three lists of candidate proteins were prepared, containing PR- and PS-specific proteins, along with proteins which were common between two groups (S4 Table).

### Identification of core lists containing hubs involved in tumor initiation and chemotherapy side effects

#### Primary analysis based on the literature and GO enrichment via Enrichr and ClueGo

**Fig 3:**
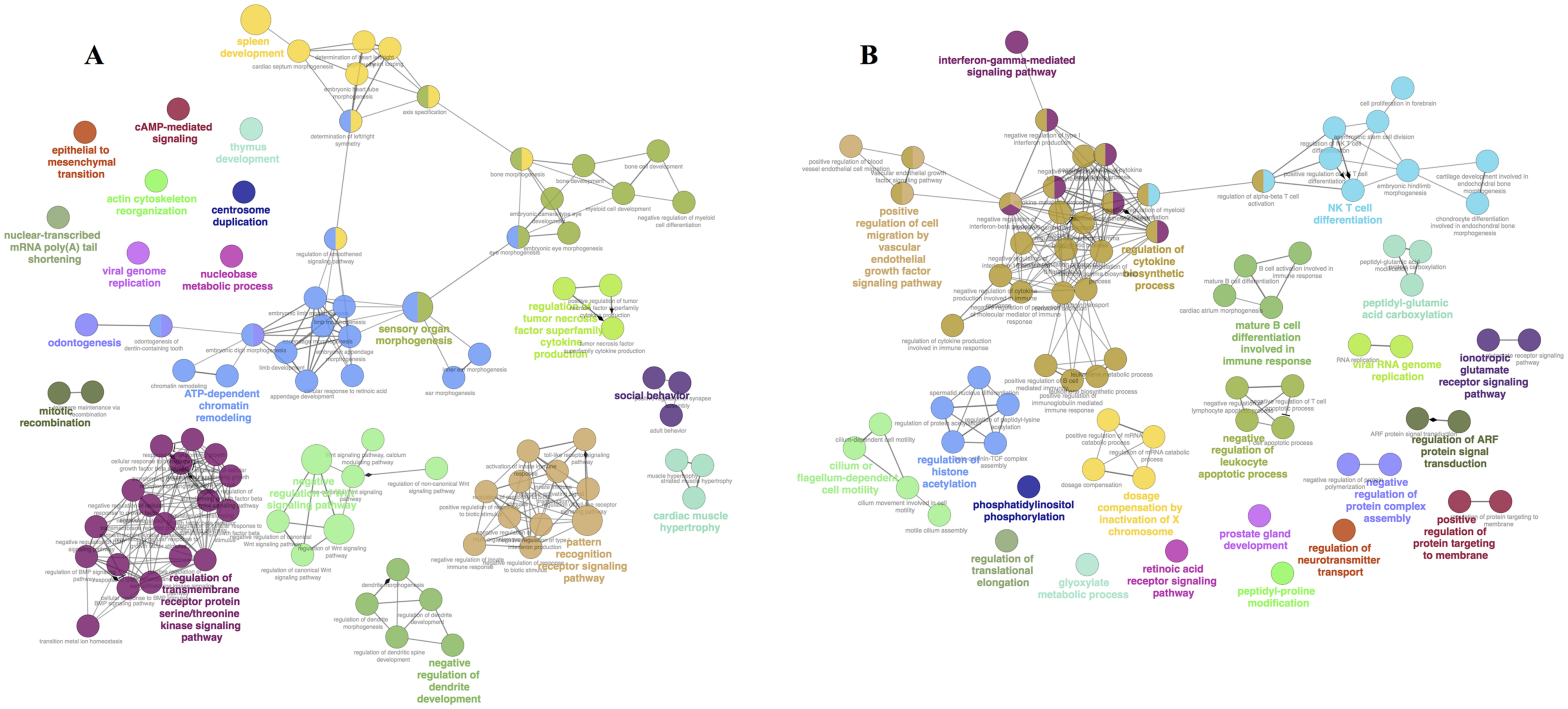
Visualization of overrepresented GO terms via Cytoscape platform based on ClueGO/CluePedia network analysis. (A) Each node represents a PR-specific term. (B) Each node represents a PS-specific term. Node size indicates *P*-value.

The GO functional analysis on PR and PS gene sets using Enrichr and ClueGo resulted in the identification of several GO terms with significant roles in cancer or NHL development, tumor progression, and/or chemotherapy side effects. The most significantly enriched GO terms in the PR group were related to biological processes such as negative regulation of Wnt SP (GO:0030178), regulation of Wnt SP (GO:0030111), hematopoietic or lymphoid organ development (GO:0048534), centrosome duplication (GO:0051298), telomere maintenance via recombination (GO: 0000722), TNF superfamily cytokine production (GO:0071706), negative regulation of myeloid cell differentiation (GO:0045638), ATP-dependent chromatin remodeling (GO:0043044), TGF-β receptor SP (GO:0007179), and regulation of BMP SP (GO:0030510) (Fig 3A). The key role of these SPs in NHL development was verified by overrepresented terms, including Wnt SP overactivity, emergence of alleviating pathways, centrosome duplication, telomere maintenance, chromatin remodeling (related to cancer cells), and events associated with spleen development.

The significant enriched terms in the PS group were related to biological processes classified in phosphatidylinositol metabolic process (GO:0046488), glutamate receptor SP (GO:0007215), regulation of cytokine biosynthetic process (GO:0042035), regulation of NKT cell differentiation (GO:0051136), interferon gamma (IFNγ)-mediated SP (GO:0060333), β-catenin-TCF complex assembly (GO:1904837), negative regulation of leukocyte apoptotic process (GO:2000107), mature B cell differentiation involved in immune response (GO:0002313), glycerophospholipid metabolic process (GO:0006650), positive regulation of immunoglobulin mediated immune response (GO:0002891), and VEGF SP (GO:0038084) (Fig 3B). The activated pathways in the PS group were associated with NHL and R-CHOP regimen, highlighting immune system-related events and adverse effects of drugs in patients who underwent chemotherapy.

#### Pathway enrichment by Enrichr, IPA, and ClueGo

In the PR group, the pathway enrichment analysis resulted in the identification of pathways, including Wnt SP, transcriptional misregulation in cancer, SPs regulating pluripotency of stem cells, IL-6 SP, and transport of nucleosides and free purine and pyrimidine bases across the plasma membrane, as repeatedly demonstrated with Enrichr (S1 Fig) and ClueGO.

The pathway enrichment analysis of PS gene set revealed high-affinity receptor for the immunoglobulin E (FcɛRI) SP, IFNγ SP, sphingolipid metabolism, Wnt/β-catenin SP in leukemia, signaling by growth factors (eg, ERBB4, PDFG, FGFRs, EGFR, and VEGFR2), CXCR4 SP, ionotropic glutamate receptor pathway, metabotropic glutamate receptor group I and III pathways, 5HT2 type receptor mediated SP, and histamine H1 receptor mediated SP (S1 Fig). These pathways associated with the pathobiology of cancers and/or the effects of chemotherapy on patients are presented in Table 1.

**Table 1:** Classification of PR and PS gene sets in a series of pathways identified through Enrichr.

	Annotation terms	The importance of pathways in cancer and chemotherapy-related events
	**KEGG**	
1	Transcriptional misregulation in cancer	Misregulation of a large amount of transcription factors, cofactors, and chromatin regulators, which direct gene expression programs, can cause various cancers [29, 30].
2	Wnt SP	Wnt SP is implicated in a variety of cancers [31–33]. There is an interaction between Notch, Akt, TGF-β, Wnt, and HIF SPs found in this study [32, 34].
3	SPs regulating pluripotency of stem cells	Wnt SP is necessary for the maintenance of cancer stem cells [35]. The key role of Wnt/β-catenin SP in regulating the differentiation of stem cell populations and the relationship between its dysregulation and numerous tumor types make this pathway an interesting target for anticancer therapeutics [36].
4	Calcium SP	Several Ca^+2^-mediated SPs are dysregulated in tumor development and progression [37]. Doxorubicin increases the intracellular Ca^+2^ level [38–40]. Rituximab-induced translocation of CD20 to lipid rafts is important in increased intracellular Ca^+2^ levels, and downstream apoptotic signaling, [41] and Cardiotoxicity*[38, 39].
5	Glioma	Secondary glioma may occur in patients due to therapy for a primary malignancy [42].
6	Fc epsilon rI (FcεRI) SP	Infiltrating mast cells induce chemotherapy resistance through activating p38/p53/p21 in cancer cells [43]. The stem cell factor enhances mast cell degranulation and cytokine production through cross-linking of FcεRI. Mast cell activation results in the secretion of histamine, serotonin, tumor necrosis factor, kinins, and proteases stored in secretory granules [44].
	**WikiPathways**	
1	IL-6 SP	In the hematopoietic system, the growth-regulatory role of IL-6 is often detected in tumors, which arise from the B cell compartment [45].
2	Sphingolipid metabolism	*De novo* synthesis and hydrolysis of sphingomyelin are often involved in ceramide generation in response to cancer therapy. Dysregulated generation of ceramide and consumption of free ceramide by incorporation into sphingomyelin (or by deacylation of ceramide to form sphingosine) are associated with defects in therapy-induced apoptosis and chemoresistance. There are several classes of cytotoxic chemotherapeutics including vincristine, doxorubicin, and topoisomerase inhibitors (irinotecan and etoposide), which can lead to ceramide accumulation [46–48].
	**Reactome**	
1	Transport of nucleosides and free purine and pyrimidine bases across the plasma membrane	Although purine nucleosides are used exclusively against hematological malignancies, pyrimidine analogs typically show efficacy against solid tumors, as well [49]. Purine nucleoside analogs, such as fludarabine, cladribine, and clofarabine are substrates for SLC29A1, SLC29A2, SLC28A3, and SLC28A2 [49]. In contrast, pyrimidine analogs, such as gemcitabine, cytarabine, and azacytidine, are transported by SLC28A1 in addition to SLC29A1, SLC29A2, and SLC28A3 [49].
2	Signaling by TGF-β receptor complex	Phosphorylation of Smad1 in TGF-β SP has been reported in NHL [50]. TGF-β contributes to both early suppression of malignancy and tumor progression in later stages [51–53]. TGF-β and Wnt SPs can synergistically promote tumorigenesis [34].
3	CREB phosphorylation through the activation of CaMKII and Ras	CaMKII is expressed at high levels in some cancers [54]. Ras/MEK/ERK SP acts as a critical pathway in cancer development and resistance to chemotherapy [55, 56].
4	Ras activation upon Ca^2+^ influx through NMDA receptor	Oncogenic mutations in a number of upstream or downstream components of Ras SP have been detected in a variety of cancers [57].
	Post NMDA receptor activation events	NMDA receptors are overexpressed in several cancers and play important roles in proliferation of cancer cells [58]. Overactivity of NMDA receptors is correlated to apoptotic neuronal damage [59]. One of the adverse effects of doxorubicin on normal cells is neurotoxicity due to the induction of apoptosis in neural cells§ [60, 61].
6	Gamma-carboxylation of protein precursors	Venous thromboembolism (VTE) is a frequent and potentially fatal complication associated with hematological and solid tumor malignancies. In patients with cancer, the occurrence of VTE is an indicator of poor prognosis. The annual incidence of VTE in patients on chemotherapy is estimated at 11%, which can rise to 20% or higher, depending on the type of drug(s) being used [62].
7	VEGFR, FGFRs, and ERBB4	The increased level of growth factors and their receptors (eg, VEGFR, FGFRs, and ERBB4) is associated with tumor formation and drug resistance [63–66].
8	IFNγ signaling	Doxorubicin induces IFN-responsive genes via IFNγ-JAK-STAT1 SP, leading to doxorubicin cytotoxicity [67]. The cellular response to DNA damage is activation of IFN signaling [67].
	**BioCarta**	
1	CXCR4 SP	The function of CXCL12/CXCR4 is essential for homing and/or engraftment of hematopoietic stem cells (HSCs) to the bone marrow after transplantation. Treatment of NHL patients with plerixafor (an antagonist of alpha CXCR4) and G-CSF caused an increase in the number of HSCs used for autologous transplantation [68].
	**Panther**	
1	Metabotropic glutamate receptor group I and III pathways	mGlu receptors are as novel targets for the treatment of aggressive or chemotherapy-resistant tumors [69]. Tumors secreting glutamate are highly resistant to chemotherapy and standard apoptosis-inducing therapeutics [70].
2	Oxytocin receptor mediated SP	Oxytocin receptor is in the cluster of overexpressed genes related to doxorubicin resistance [71].
3	5HT-2 type receptor mediated SP	Dysregulation of central 5HT metabolism or function may be a contributing factor in chemotherapy-induced nausea and vomiting, and cancer-related fatigue [72–74].
4	Histamine H1 receptor mediated SP	Mast cell activation results in histamine release and diverse side effects [75]. High amounts of histamine as well as histamine receptors have been observed in different cancers [76]. A dose of doxorubicin (1 mg/kg) can lead to histamine and catecholamines release, producing the cardiomyopathy in dogs [77].

The effects of PR- and PS-related pathways in different cancers and chemoresistance are presented based on the KEGG, WikiPathways, Reactome, BioCarta, and Panther databases (white rows, PR; red rows, PS; and yellow rows, calcium SP as a common pathway between the groups). *P*-value less than 0.05 was considered statistically significant.

*Myofibrillar deterioration and intracellular calcium dysregulation are important mechanisms commonly associated with doxorubicin-induced cardiac toxicity. Doxorubicin-induced cardiotoxicity is also accompanied by an increase in the intracellular calcium levels.

^§^Glucocorticoids exert a significant protection against NMDA-induced neuronal necrosis, at least in part via their ability to enhance glutamine synthetase in glial cells.

**Abbreviations**:

BDNF: Brain-derived neurotrophic factor, CaMKII: Ca^2+^/calmodulin-dependent protein kinase II, ERBB4: Receptor tyrosine-protein kinase erbB-4, FGFRs: Fibroblast growth factor receptors, mGlu receptors: metabotropic glutamate receptors, NGF: Nerve growth factor, NMDAR: N-methyl-D-aspartate receptor, SP: Signaling pathway, and VEGFR: Vascular endothelial growth factor.

In the present study, there were common pathways between the PR and PS groups, including calcium SP with different proteins in the PR (TNRC6C, FZD6, AXIN2, and NLK) and PS (GRM5, CAMK2A, PLCG2, CACNA1A, CACNA1S, PLCG1, and GRIN2D) groups, Wnt/β-catenin SP (PR: ANKRD6, AXIN2, CHD8, FZD6, HDAC1, HECW1, NKD1, NKD2, NLK, RNF213, and RNF43; and PS: PYGO1 and ZBTB16), and inositol phosphate metabolism with three common proteins (ie, PIK3C2G, MTMR4, and MTMR6). Furthermore, two pathways were significantly correlated with drugs such as G-CSF and erythropoietin used in the PS group.

S2 Fig shows the top canonical pathways in the PR and PS groups identified via IPA. The top five canonical pathways identified in the PR gene set were transcriptional regulatory network in embryonic stem cells (ESCs), RhoA signaling, adipogenesis pathway, telomerase signaling, and DNA methylation and transcriptional repression signaling (S5 Table). Other significant pathways in this group were nuclear receptor 4A1 (Nur77) signaling in T lymphocytes and Wnt/β-catenin SP. Enriched canonical pathways found in the PS gene set were neuropathic pain signaling in dorsal horn neurons, synaptic long-term potentiation, dopamine-DARPP32 feedback in cAMP signaling, neuronal nitric oxide synthase (nNOS) signaling in neurons, and FcɛRI signaling (S6 Table). Other remarkable pathways in this group were leukocyte extravasation signaling, glioma signaling, ERBB4 signaling, and chemokine signaling, as well as Wnt/β-catenin SP which was also observed in the PR group. The top three upstream regulators in the PR gene set were SOX2 (*P*-value: 1.49E-05), SOX2-OCT4-NANOG (*P*-value: 3.95E-05), and NANOG (*P*-value: 4.16E-04). Additionally, ZEB2 (*P*-value: 9.51E-04) was found as one of the top upstream regulators associated with the PS gene set. In both PR and PS groups, cancers, as well as organizmal injury and abnormalities [*P*-value _PR_: 1.88E-02–1.00E-12 (n = 190, 93%); *P*-value _PS_: 2.48E-02–5.65E-10 (n = 165, 91%)], and gastrointestinal disease [*P*-value _PR_: 1.85E-02–2.18E-10 (n = 170, 83%); *P*-value _PS_: 1.98E-02–7.44E-08 (n = 146, 81%)] were the top three IPA-predicted diseases and disorders. Moreover, evaluation of two gene lists in the disease association analysis of WebGestalt database exhibited disorders, such as chromosome aberrations, leukemia, genetic translocation, and bone neoplasms in the PR group, as well as dysarthria, schizophrenia, eosinophilia, acquired immune deficiency syndrome-related complex, and lentivirus infections in the PS group (*P* <0.05).

#### Analysis of modules and hubs extracted from protein-protein interaction networks

**Fig 4:**
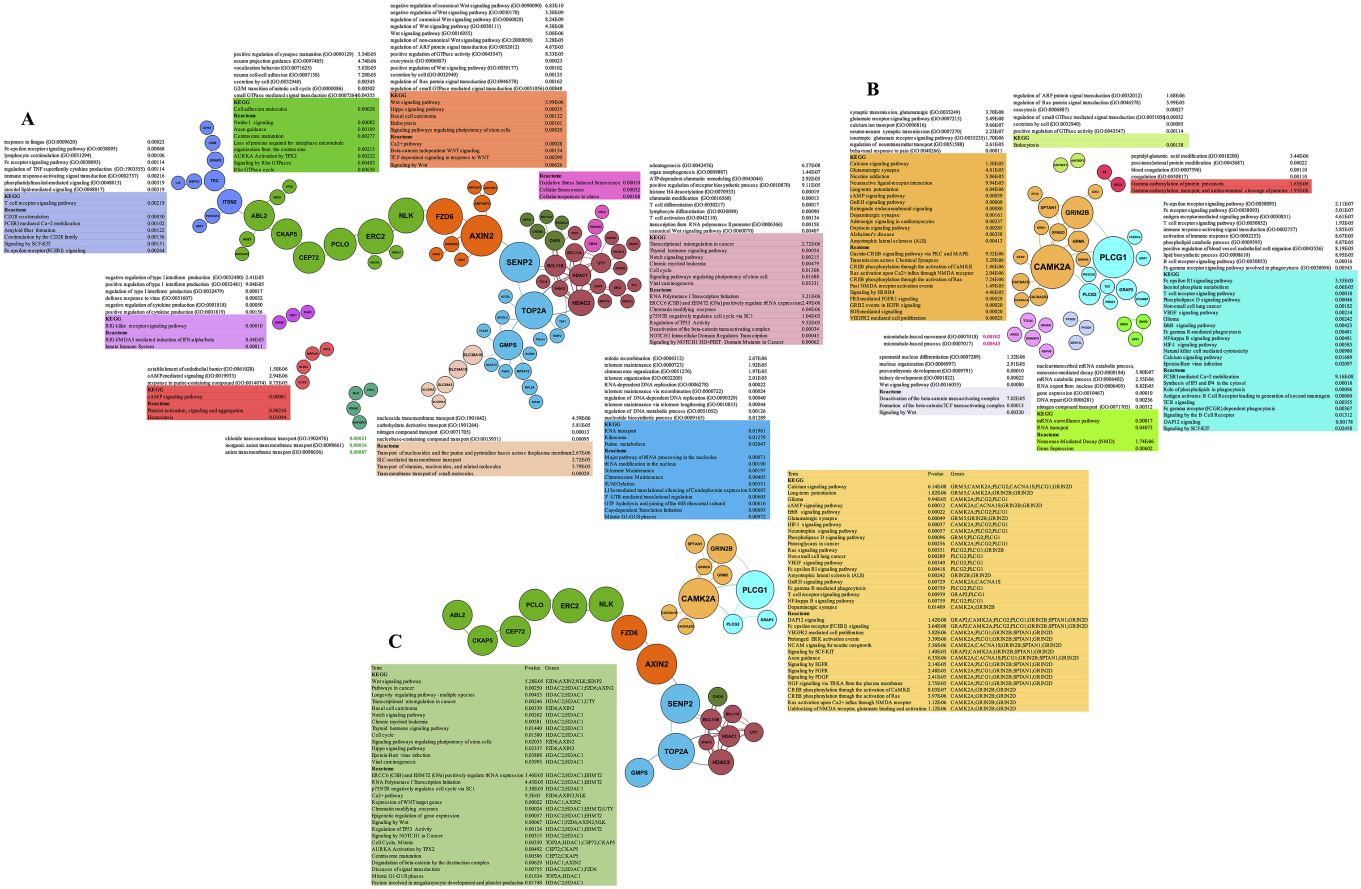
The GO and pathway analysis of modules and hubs obtained from STRING and Gephi. (A) The nodes are PR-specific proteins arranged according to betweenness centrality and colored in different modules based on the fast unfolding clustering algorithm implemented in Gephi [25]. (B) The nodes are PS-specific proteins arranged according to betweenness centrality and colored in different modules. (C) The nodes are PR- and PS-specific hubs. GO terms and significant pathways related to the modules as well as significant pathways associated with hubs are listed according to Enrichr in each group (based on the KEGG and Reactome databases).

To reveal the key proteins/hubs in each biological function or pathway, protein-protein interactions in the PR and PS groups were extracted from STRING and then visualized via Gephi. As shown in Fig 4A and 4B, PR-related protein-protein interaction network (PPIN) contained 85 nodes and 103 edges; in contrast, PPIN of PS proteins included 37 nodes and 46 edges. In each PR- and PS-related PPIN, the nodes sizes were sorted based on the term of betweenness centrality and were represented in the modules with different colors. For determining the functionality of the networks, GO and pathway enrichment analysis of each module in the PR and PS networks were carried out via Enrichr, which demonstrated that the overrepresented terms were in harmony with the results observed in previous sections. Indeed, the conformity of enriched terms in a module with other detected terms in the present study verified the value of module-related results.

Table 2 shows the high degree nodes and their betweenness centrality in the PR and PS groups. The pathway enrichment analysis of PR- and PS-specific hubs was carried out through Enrichr (based on the KEGG and Reactome databases) (Fig 4C). The analysis exhibited that PR-specific hubs were associated with statistically significant pathways such as Wnt SP, transcriptional misregulation in cancer, ERCC6 (CSB), and EHMT2 (G9a) positively regulate rRNA expression, and chromatin modifying enzymes involved in tumor formation and development.

**Table 2:** The PR and PS protein core lists.

	Hub	Degree	Betweenness centrality	Drugs targeting hub
1	AXIN2	5	**1346**	
2	SENP2	4	**1294**	
3	TOP2A	***8***	**1156.83**	Amsacrine, Valrubicin, Teniposide, Etoposide, Doxorubicin, Idarubicin, Mitoxantrone, Epirubicin, and Podofilox
4	FZD6	3	**1092.0**	
5	ERC2	4	**1061**	
6	NLK	2	**1060**	
7	PCLO	2	912	
8	CEP72	2	870	
9	CKAP5	2	826	
10	ABL2	4	820	Adenosine triphosphate and Dasatinib
11	GMPS	***8***	779.5	
12	HDAC2	***10***	601	Vorinostat, Panobinostat, and Romidepsin
13	HDAC1	***9***	391	Vorinostat, Panobinostat, and Romidepsin
14	BCL11A	***6***	372	
15	UTY	***8***	282.5	
16	CHD5	5	281	Epirubicin (Targets CHD1)
17	BCL11B	4	225	
18	EHMT2	***6***	112	
1	CAMK2A	***9***	**97.83**	
2	PLCG1	***9***	**90**	
3	GRIN2B	***7***	**58.66**	
4	PLCG2	***6***	22.66	
5	GRM5	4	20	
6	GRAP2	4	20	
7	SPTAN1	3	20	
8	CACNA2D3	2	9.5	
9	CACNA1S	2	9.5	
10	GRIN2D	5	9.3	

PR- and PS-specific hubs (red and blue, respectively) are classified according to their degree, betweenness centrality, and drugs targeting them. The hubs with high betweenness centrality are shown in bold and high degree hubs are presented in bold and italic.

Calcium SP, long-term potentiation, glioma, ERBB SP, FcɛRI mediated MAPK activation, unblocking of an NMDA receptor, glutamate binding and activation, as well as VEGFR2 mediated cell proliferation were the enriched pathways based on the pathway analysis of PS hubs. Indeed, these pathways reflected events such as side effects of drugs and cancer recurrence in patients who received chemotherapy.

Furthermore, the hubs were classified according to their alterations and involvements in various cancer pathways. Among 18 and 10 hubs in the PR and PS groups, respectively, nearly half of them were classified in the high expression group (n_PR_ = 10; n_PS_ = 4), while others were categorized in the mutation (n_PR_ = 3; n_PS_ = 3) and the translocation (n_PR_ = 2) groups (Table 3). Through mining approximately 400 articles, we evaluated hubs in pathways and occurrences that have critical roles in cancers. As shown in Table 4, there were considerable relationships between the detected hubs and events related to tumor progression such as cell cycle and DNA repair (TOP2A, HDAC1, HDAC2, BCL11A, BCL11B, POLA1, EHMT2, CEP72, GRM5, CAMK2A, GRAP2, and CACNA2D3), HIF-1α and VEGF production (PLCG and GRM5), chemoresistance (PLCG2 and PLCG1), chemotherapy outcomes (GRM5 and SPTAN1), and even autophagy (EHMT2).

**Table 3:** Literature based classification of PR- and PS-specific hubs.

Alterations in cancers (high expression, mutation, and translocation)				
Cancer	High expression (or gene amplification)	Mutation (and/or polymorphism)	Translocation	Diverse
**Lymphoma**	**TOP2A**[78, 79], **BCL11A**[80], **BCL11B**[81], and *PLCG2*[82]	*PLCG1*[83]	**BCL11A**[84]	**BCL11B**[85]
**Other cancers**	**AXIN2**[86, 87], **NLK**[88], **TOP2A**[89], **HDAC1** & **HDAC2**[90], **BCL11A**[80, 84], **BCL11B**[85, 91], **EHMT2**[92], **FZD6**[93, 94], *PLCG2*[82], *PLCG*[95, 96], *GRM5*[97, 98], and *GRAP2*[99]	**AXIN2**[86], **CAMKK1**[100], **CEP72**[101]*, *GRIN2D*[102, 103], and *GRIN2B*[70]	**BCL11A**[84] and **BCL11B**[91]	**CKAP5**[104], **CHD5**[105–108], *GRIN2B*[109], and *CACNA2D3*[110, 111]

Hubs were grouped based on their alterations in lymphoma and/or other cancers. The diverse group refers to the alterations other than overexpression, mutation, and translocation. PR- and PS-specific hubs are presented in bold and italic, respectively.

*Polymorphism in promoter region.

**Table 4:** Literature based classification of PR- and PS-specific hubs.

Pathways and programs	Hubs	
**Wnt/β-catenin SP**	**TOP2A**[112], **EHMT2**[113, 114], **AXIN2**[86, 87], **FZD6**[115], **NLK**[88, 116], and **SENP2**[117]	
**P53 networks**	**HDAC1**[90], **SENP2**[118], and **CHD5**[108, 119]	
**Progression**	CSC	*PLCG1*[95]
	EMT programs	**HDAC1**, **HDAC2**[120], **CHD5**[108], *PLCG1*[95], *GRM5*[121], *SPTAN1*[122], and *CACNA2D3*[111]
	HIF-1α and VEGF production	*PLCG*[123] and *GRM5*[121]
**Chemotherapy**	Resistance	*PLCG2*[124] and *PLCG1*[95]
	Drug effects	*GRM5*[97] and *SPTAN1*[125]
**Cell cycle and DNA repair (proliferation)**	**NLK**[88], **AXIN2**[126], **TOP2A**, **HDAC1**, **HDAC2** [90], **CEP72**[127], **BCL11A**[84], **BCL11B**[85], **POLA1**, **EHMT2**[128], **CHD5**[108], *GRM5*[98], *CAMK2A* [129], *GRAP2*[130], and *CACNA2D3*[111]	
**Apoptosis**	**HDAC2**[90], **BCL11B**[81], *SPTAN1*[131], and *CACNA2D3*[111]	
**Autophagy**	**EHMT2**[132]	
**Diverse**	**CKAP5**[133], **CEP72**[101], **UTY**, *GRIN2D*[103], *GRIN2B*, and *CACNA1S*	

Hubs were grouped based on their contributions to different pathways involved in cancer pathogenicity and chemotherapy-related events. Hubs which are not involved in significant pathways and programs, are included in the diverse group. PR- and PS-specific hubs are presented in bold and italic, respectively.

**Abbreviations**:

CSC: Cancer stem cell and EMT: Epithelial-mesenchymal transition.

The evaluation of PR and PS hub genes in the disease association analysis of WebGestalt database revealed disorders, which are certainly connected to the related group. Cancer or viral infections, lymphoid leukemia NOS, and B-cell lymphoma were significant diseases associated with PR-specific hubs. However, acquired immune deficiency syndrome-related complex, schizophrenia, mental disorders, depression, HIV, and dementia were correlated with PS specific-hubs (*P* < 0.05).

#### Interactions between drugs and PR- and PS-specific proteins

The assessment of PR-specific hubs in the DrugBank database led to the identification of hubs which were targets for chemotherapy agents being used in NHL patients. Table 2 shows the approved drugs inhibiting HDAC1, TOP2A, and ABL2. Considering the emergence of these targets involved in tumor formation in the PR group, in addition to the effectiveness of drugs targeting them, other hubs in this list may be novel targets which can be used to find effective drugs. In comparison with the PR group, targets such as TOP2A and the other abovementioned proteins were not observed again in the PS group. PS-specific proteins were mostly involved in the outcomes of chemotherapy agents, including various members of solute carrier family (S4 Table) which may be associated with other members of this family such as SLC22A16, SLC22A3, and SLCO1A2 that are doxorubicin, vincristine, and prednisolone transporters, respectively. In the solute carrier family, SLCO6A1 was found in the PR group, as well.

#### The aptitude of SENP2 and PLCG1 for discrimination of NHL patients and healthy controls

**Fig 5:**
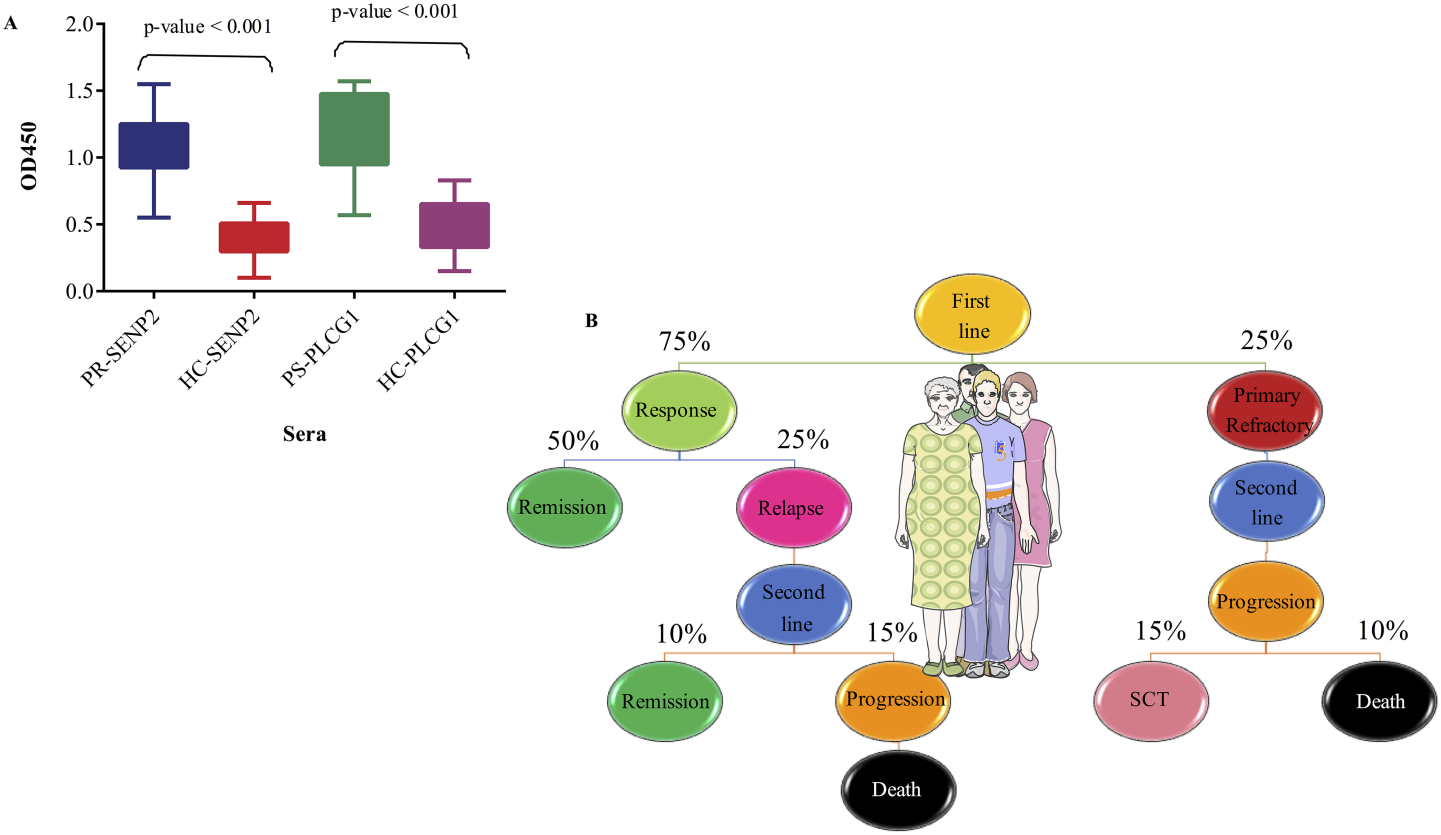
Legitimacy of AAb repertoires for discrimination of NHL patients and healthy subjects through two highly ranked hubs and follow-up of NHL patients after first- and second-line chemotherapy regimens. (A) The significant binding of SENP2 and PLCG1 to the sera of 10 PR (blue) and 20 PS (green) patients in comparison with the sera of age-matched healthy subjects (HC) (red and purple, respectively) verifies the accuracy of identified hubs in the NHL groups. (B) Flow of 20 PS patients followed-up every three months for the first two years after treatment and then every six months using physical examination and relevant laboratory tests (SCT: autologous or allogeneic stem cell transplantation).

Based on features such as degree and betweenness centrality, SENP2 and PLCG1 were selected in the PR and PS groups, respectively. To assess the capacity of these hubs for discrimination of NHL patients and healthy controls, the sera of 10 patients from the PS group, 20 patients from the PR group, and 30 age-matched healthy controls were used for validation. The data revealed specific binding of SENP2 and PLCG1 to the sera of PR and PS patients, respectively (*P* < 0.001). In contrast, they exhibited weak to moderate binding to the sera of age-matched healthy subjects. Their moderate signal intensities might be due to cross-reactivity between two hubs (SENP2 and PLCG1) and large amounts of proteins in the sera of healthy controls (Fig 5A).

### Unfavorable relapse/refractory NHL in a significant proportion of PS patients according to the two-year follow-up

Twenty patients of from the PS group were followed-up to evaluate events which occurred in patients over two years during or after chemotherapy. Fifteen cases showed a response to the first-line treatment, including an anthracycline-containing regimen with or without rituximab (75%). Among these patients, 10 (50%) experienced complete remission, while five (25%) with relapse underwent second-line chemotherapy. Three patients from the latter group showed progression under therapy and passed away, while the remaining two patients showed complete response to treatment and experienced remission.

Among five patients with primary refractory NHL (25%), two revealed progression under second-line chemotherapy and passed away, whereas the remaining three patients, given their response to treatment, were candidates for high-dose therapy and autologous or allogeneic stem cell transplantation (Fig 5B).

## Discussion

Treatment complications in patients with NHL signify an unmet need for developing novel therapies in both first-line and relapse settings [134]. Accordingly, AAb signatures have been recently used to reveal the identity of antigens involved in events related to disease severity, cancer relapse, and treatment response [10]. Notably, AAbs are not only detectable long before the clinical manifestation of a tumor appears, but also persist for a prolonged period, even if the corresponding autoantigens are no longer apparent [9, 10].

Various studies have reported a panel of AAbs in patients with different cancers, as well as NHL [9, 11, 12]. In different studies on patients with NHL, evaluation of their AAb repertoires, led to the identification of a set of AAbs, such as anti-histidyl-tRNA synthetase antibody (anti-Jo-1), cytoplasmic antineutrophil cytoplasmic antibody (c-ANCA), antinuclear antibody (ANA), rheumatoid factor, anti-topoisomerase I antibody (anti-Scl-70), antiphospholipid antibody (APA), and anti-single-stranded DNA antibody (anti-ssDNA) [11, 12, 135, 136]. Although the majority of generated AAbs are against nuclear antigens, AAbs against peripheral-nerve antigens have also been detected in NHL patients [11].

In harmony with several studies, we not only found a previously reported panel of AAs targeting proteins, but also identified AAbs against proteins which had not been reported in NHL patients. The occurrence of somatic hypermutation in germinal center (GC) B cells which are majorly involved in several NHL subtypes, leads to the emergence of GC B cells with high affinity for self-antigens. These cells receive more survival signals and differentiate into plasma cells or memory B cells which can identify autoantigens easier than normal B cells and persistently generate AAbs after several years [137].

In this study, AAbs were generated against PR- and PS-specific proteins as intra- or extra-cellular proteins showing different alterations, including overexpression, mutation, translocation, and PTM. They were then exposed as foreign antigens to immune cells due to the high rate of proliferation and defects in the cellular death mechanism of cancer cells.

In parallel to various studies which have demonstrated the most important pathways and events such as Wnt SP, transcriptional misregulation in cancer, Notch SP, and telomere maintenance in overactivated cancer cells, we found similar pathways in the PR group. Moreover, we identified pathways which were either linked to NHL-related events (eg, spleen development and activation of different immune cells) or triggered in response to overactivated pathways in cancer cells (eg, negative regulation of canonical Wnt SP).

Additionally, a set of interesting hubs was identified, some of which such as AXIN2, SENP2, TOP2A, FZD6, NLK, CEP72, CKAP5, HDAC2, HDAC1, UTY, and EHMT2 were involved in the mentioned PR-related pathways. As drugs such as doxorubicin, etoposide, and mitoxantrone have been designed to target TOP2A in cancer patients, other hubs of this group may also have the potential as functional therapeutic targets to modify targeted therapy outcomes [138]. Along these lines, there are several drugs such as vorinostat and depsipeptide which inhibit HDAC and agents such as olokizumab and raloxifene which have been designed to bind to IL6ST and block IL-6 SP [139, 140]. These agents are under investigation in a variety of clinical trial phases for different cancers and can be exploited to evaluate in NHL [139, 140].

In the PS group, the most commonly identified pathways were related to processes triggered after treatment, which induced chemotherapy side effects and tumor development in a manner different from the PR group. The most important PS-related pathways in this study were calcium- and glutamate-related SPs. According to the literature, chemotherapeutic agents can lead to peripheral sensitization by up regulation of NMDA receptors (NMDARs) and protein kinase C. This can help determine why AAbs were generated against two subunits of NMDARs and a number of proteins in calcium SP [141]. In addition, chemotherapeutic agents generate reactive oxygen species (ROS) which inactivate SLC1A2. As a result, glutamate transporters are disturbed, and excessive activation of NMDARs by glutamate leads to an excitatory event and neurotoxicity [142, 143]. Notably, expression of NR1, NR2B, and NR2D subunits of NMDARs has been reported in different cancer cells [144].

One type of toxicity with particular importance in cancer patients is chemotherapy-induced peripheral neuropathy (CIPN) which can lead to permanent symptoms and disability in approximately 40% of cancer survivors [145]. The most common mechanisms involved in CIPN are nuclear DNA damage, microtubule changes, ROS production, mitochondrial function impairment, calcium signaling changes, and disturbances in glutamate signaling which are all completely associated with pathways identified in our PS patients [146].

The emergence of relapse and death in our patients who underwent chemotherapy revealed the involvement of pathways in cancer progression and relapse. These pathways can affect treatment outcomes, and some of them can awaken dormant tumor cells, resulting in cancer recurrence after chemotherapy. As the results showed, a set of hubs such as CAMK2A, PLCG1, GRIN2B, PLCG2, GRM5, GRAP2, SPTAN1, CACNA2D3, CACNA1S, and GRIN2D in the PS group were involved in FcɛRI SP, FcγR dependent phagocytosis, DAP12 signaling, calcium SP, HIF-1 SP, phospholipase D SP, and various growth factor-related SPs (VEGF, FGFRs, PDGF, and ERBB4) which are associated with R-CHOP regimen and chemoresistance [68, 147–149].

The present study had several limitations. Although evaluation of 10 nontreated patients could provide remarkable data, the sample size recruited in this study was limited. Furthermore, it seems reasonable to validate the findings in an independent cohort study, as we only verified the presence of AAbs against the selected hubs in the recruited patients. Considering these limitations, we present PR- and PS-specific hubs and their related pathways are mostly associated with tumor cell growth, flanking of the immune system, and treatment effects. These hubs may be functional if used as biomarkers and/or therapeutic targets for determining the best treatment strategy and designing novel drugs for patients who have not initiated treatment or have undergone first-line chemotherapy without any promising results.

## Supporting information

S1 Text Detailed descriptions of phage ELISA, b‌‌inding of the selected hubs to the sera of PR and PS patients, and follow-up of NHL patients who underwent chemotherapy. (DOCX)

S1 Fig Pathways identified through Enrichr. (A) The PR gene signature and (B) PS gene signature have been classified in a set of pathways and ordered according to *P*-value by mining in the databases, including KEGG (pink), WikiPathways (lilac), Reactome (blue), BioCarta (green), NCI-Nature (grey), and Panther (orange) in Enrichr.
(PDF)

S2 Fig Pathway analysis through IPA. (A) PR-related pathway. (B) PS-related pathway. *P* < 0.05 is considered statistically significant.
(PDF)

S1 Table Baseline characteristic of NHL patients. (DOCX)

S2 Table Baseline characteristic of healthy volunteers. (DOCX)

S3 Table List of peptides identified through panning on the purified IgG of PR (FNs) and PS (FTs). FN7, FT2, and FT4 have similar amino acid sequences (orange). Four peptides FN2 & FN8 (pink) and FT1 & FT9 (blue) were selected for verification by ELISA.
(DOCX)

S4 Table List of proteins predicted from PR- and PS-specific peptides. The cut-off maximum score for selecting proteins in the Refseq database of BLASTP was considered equal or more than 18.5. Proteins with score more than 18.5 were nominated to investigate in more detail. (A) List of predicted PR proteins, (B) list of predicted PS proteins, and (C) list of proteins which were common between two groups.
(DOCX)

S5 Table Top canonical networks at PR gene signature identified through IPA. (A) Transcriptional regulatory network in embryonic stem cells (Ratio: 4/40 and *P*-value: 5.42E-04), (B) RhoA signaling (Ratio: 6/122 (0.049) and *P*-value: 1.08E-03), (C) Adipogenesis pathway (Ratio: 4/134 (0.037) and *P*-value: 8.97E-03), (D) Telomerase signaling (Ratio: 4/99 (0.04) and *P*-value: 1.45E-02), and (E) DNA methylation and transcriptional repression signaling (Ratio: 2/20 (0.1) and *P*-value: 1.51E-02). N: Nucleus, TR: Transcription regulator, C: Cytoplasm, E: Enzyme, PM: Plasma membrane, GPCR: G-protein coupled receptor, P: Phosphatase, and PP: Peptidase.
(DOCX)

S6 Table Top canonical pathways at PS gene signature identified through IPA. (A) Neuropathic pain signaling in dorsal horn neurons (Ratio: 7/100 (0.07) and *P*-value: 2.07E-05) and synaptic long term potentiation (Ratio: 7/119 (0.059) and *P*-value: 6.34E-05), (B) CREB signaling in neurons (Ratio: 8/171 (0.047) and *P*-value: 9.63E-05), (C) Dopamine-DARPP32 feedback in cAMP signaling (Ratio: 7/161 (0.043) and *P*-value: 4.12E-04), and (D) nNOS signaling in neurons (Ratio: 4/47 (0.085), *P*-value: 6.30E-04). nNOS: Neuronal nitric oxide synthase, TR: Transcription regulator, C: Cytoplasm, E: Enzyme, PM: Plasma membrane, K: Kinase, GPCR: G-protein coupled receptor, and IC: Ion channel.
(DOCX)

## Abbreviations

AAbs: autoantibodies
ABL2: Abelson tyrosine-protein kinase 2
ANKRD6: Ankyrin repeat domain-containing protein 6
BCL11A & B: B-cell lymphoma/leukemia 11A & B
BMP: Bone morphogenetic proteins
CACNA1S: Voltage-dependent L-type calcium channel subunit alpha-1S
CACNA2D3: Voltage-dependent calcium channel subunit alpha-2/delta-3
CAMK2A: Calcium/calmodulin-dependent protein kinase type II subunit alpha
CEP72: Centrosomal protein of 72 kDa
CHD5: Chromodomain-helicase-DNA-binding protein 5
CHD8: Chromodomain-helicase-DNA-binding protein 8
CKAP5: Cytoskeleton-associated protein 5
EGFR: Epidermal growth factor receptor
EHMT2: Histone-lysine N-methyltransferase EHMT2
ELISA: Enzyme-linked immunosorbent assay
ERBB: Receptor tyrosine-protein kinase erbB
ERC2: ERC protein 2
FGFRs: Fibroblast growth factor receptors
FZD6: Frizzled-6
GMPS: GMP synthase [glutamine-hydrolyzing
GRIN2B: Glutamate receptor ionotropic, NMDA2B
GRIN2D: Glutamate receptor ionotropic, NMDA 2D
GRM5: Metabotropic glutamate receptor 5
GRAP2: GRB2-related adapter protein 2
HDAC1 & 2: Histone deacetylase 1 & 2
HECW1: E3 ubiquitin-protein ligase HECW1
HIF-1α: Hypoxia-inducible factor 1-alpha
IgG: Immunoglobulin G
IL-6: Interleukin 6
MTMR4 & 6: Myotubularin-related protein 4 & 6
NHL: Non-Hodgkin lymphoma
NKD1 & 2: Protein naked cuticle homolog 1 & 2
NKT: natural killer T
NLK: Serine/threonine-protein kinase NLK
NMDA: N-methyl-D-aspartate
NOS: Not otherwise specified
PCLO: Protein piccolo
PDFG: Platelet-derived growth factor
PIK3C2G: Phosphatidylinositol-4-phosphate 3-kinase catalytic subunit type 2 gamma
PLCG1 & 2: Phospholipase C, gamma 1 & 2
PYGO1: Pygopus homolog 1
RNF43: E3 ubiquitin-protein ligase RNF43
RNF213: E3 ubiquitin-protein ligase RNF213
ROS: Reactive oxygen species
SENP2: Sentrin-specific protease 2
SLC22A16 & 3: Solute carrier family 22 member 16 & 3
SLCO1A2: Solute carrier organic anion transporter family member 1
SPTAN1: Spectrin alpha chain, non-erythrocytic 1
TNF: Tumor necrosis factor
TNRC6C: Trinucleotide repeat-containing gene 6C
TOP2A: DNA topoisomerase 2-alpha
UTY: Histone demethylase UTY
VEGF: Vascular endothelial growth factor
ZBTB16: Zinc finger and BTB domain-containing protein 16
