# Supplementary material for: A systems medicine approach for finding target proteins affecting treatment outcomes in patients with non-Hodgkin lymphoma
Source: PLoS One. 2017 Sep 11;12(9):e0183969. doi: 10.1371/journal.pone.0183969 (PMC5593188; doi:10.1371/journal.pone.0183969)
Supplement: S1 Text — (DOCX) [file pone.0183969.s001.docx]

**Supporting information (S1 text)**

**Detailed descriptions of phage ELISA, b‌‌inding of the selected hubs to the sera of PR and PS patients, and follow-up of NHL patients who underwent chemotherapy.**

**Phage ELISA**

To evaluate the specificity of phages to the PR and PS IgG, polyclonal and monoclonal phage ELISA assays were done according to the manufacturer's instructions (Ph.D.TM-C7C Kit). In brief, following blocking, incubation, and several times of washing with PBST (0.5% Tween-20 in phosphate buffered saline (PBS)), the isolated phages from three rounds of panning mentioned as inputs and outputs were added into the wells coated with the PR and PS IgG as well as empty wells as negative controls. After washing, bound phages were detected using HRP-conjugated anti-M13 antibody (GE Healthcare, 1:7000 in 0.1 M NaHCO3, 5 mg/ml BSA), and incubated for 1 h at 37 °C. Following washing, color development was stopped by adding HCl solution. The absorbance value of each well at optical density (OD) of 450 nm was measured by microplate spectrophotometer (Epoch, BioTek). The highest signals were exhibited in the input and output of third round of panning done on purified PR and PS IgGs. Thirty clones of each group were randomly selected from the third round of panning and examined by monoclonal phage ELISA as was mentioned in polyclonal phage ELISA. Eleven phage clones of each NHL group that showed higher intensities in comparison with the control were selected for DNA sequencing.

**B‌‌inding of the selected hubs to the sera of PR and PS patients**

Two 96-well plates were coated separately with SENP2 and PLCG1 recombinant proteins (100ng/well) and incubated at 4 ^o^C for overnight. Following washing and blocking with skimmed milk in PBS, 10, 20, and 30 serum samples of the PR group, the PS group, and age-matched healthy subjects (HC) diluted in PBS (1 mg/ml), were added to the related wells, respectively. After several times washing with PBST, the wells were incubated with goat anti-human IgG antibody conjugated HRP (Abcam) for 1 h at room temperature (RT). Following several washings with PBST, reactions were developed by TMB and stopped by HCL. The cutoff level for positive reactions was calculated according to means plus 2 SDs of the OD_450_ readings of the healthy control.

**Follow-up of NHL patients who underwent chemotherapy**

The PS patients enrolled in this study were evaluated for 24 months and classified according to the treatment response defined as the proportion of patients with complete remission, relapse defined as the proportion of patients having disease recurrence after the achievement of complete remission, primary refractory outlined as the proportion of patients who do not respond or only partially respond to the initial therapy, as well as progression under therapy outlined as the proportion of patients with progressive disease during treatment and within three months after the treatment was finished. Assessment of response was done based on the international Workshop criteria. In this way, patients were evaluated based on physical examination, laboratory tests, computed tomography of the chest and abdomen and bone-marrow biopsy for the previous involvement by lymphoma. Follow-up was performed by referring physician every three months for the first two years after treatment and then every six months using physical examination and relevant laboratory tests [1-3].

**References**

1. Pfreundschuh M, Trümper L, Österborg A, Pettengell R, Trneny M, Imrie K, et al. CHOP-like chemotherapy plus rituximab versus CHOP-like chemotherapy alone in young patients with good-prognosis diffuse large-B-cell lymphoma: a randomised controlled trial by the MabThera International Trial (MInT) Group. The lancet oncology. 2006;7(5):379-91.

2. Zelenetz A, Hamlin P, Kewalramani T, Yahalom J, Nimer S, Moskowitz C. Ifosfamide, carboplatin, etoposide (ICE)-based second-line chemotherapy for the management of relapsed and refractory aggressive non-Hodgkin's lymphoma. Annals of Oncology. 2003;14(90001):5-10.

3. Friedberg JW. Relapsed/refractory diffuse large B-cell lymphoma. ASH Education Program Book. 2011;2011(1):498-505.
