# Supplementary figures and images for: A systems medicine approach for finding target proteins affecting treatment outcomes in patients with non-Hodgkin lymphoma
Source: PLoS One. 2017 Sep 11;12(9):e0183969. doi: 10.1371/journal.pone.0183969 (PMC5593188; doi:10.1371/journal.pone.0183969)

A

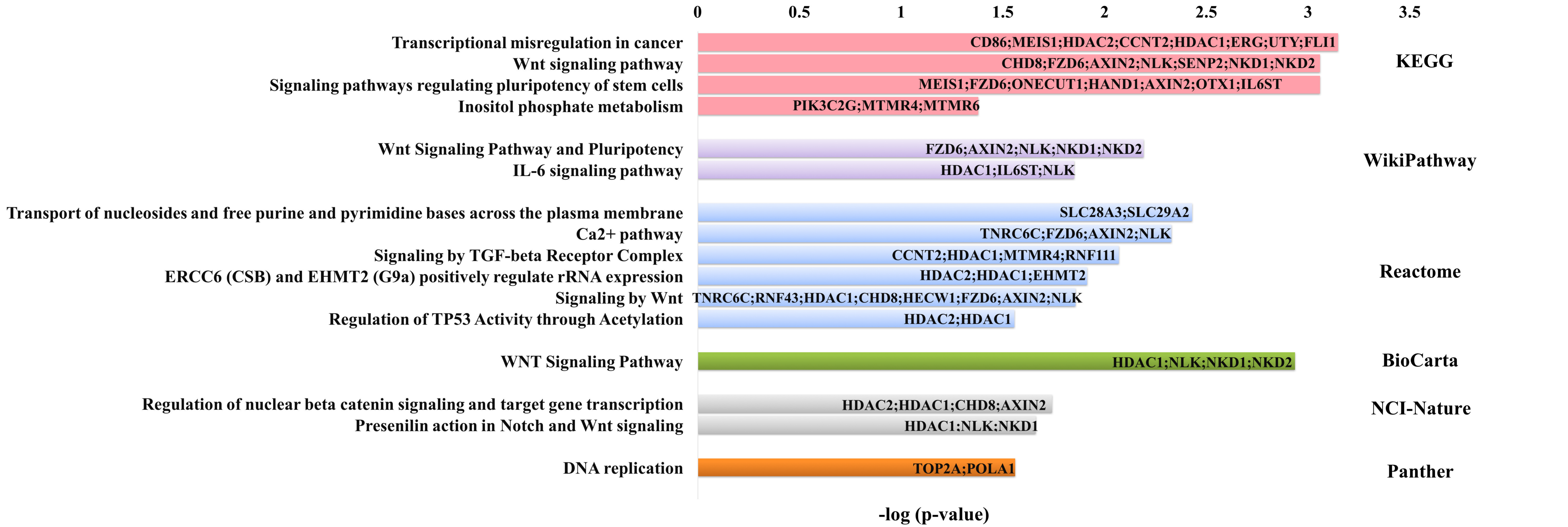

B

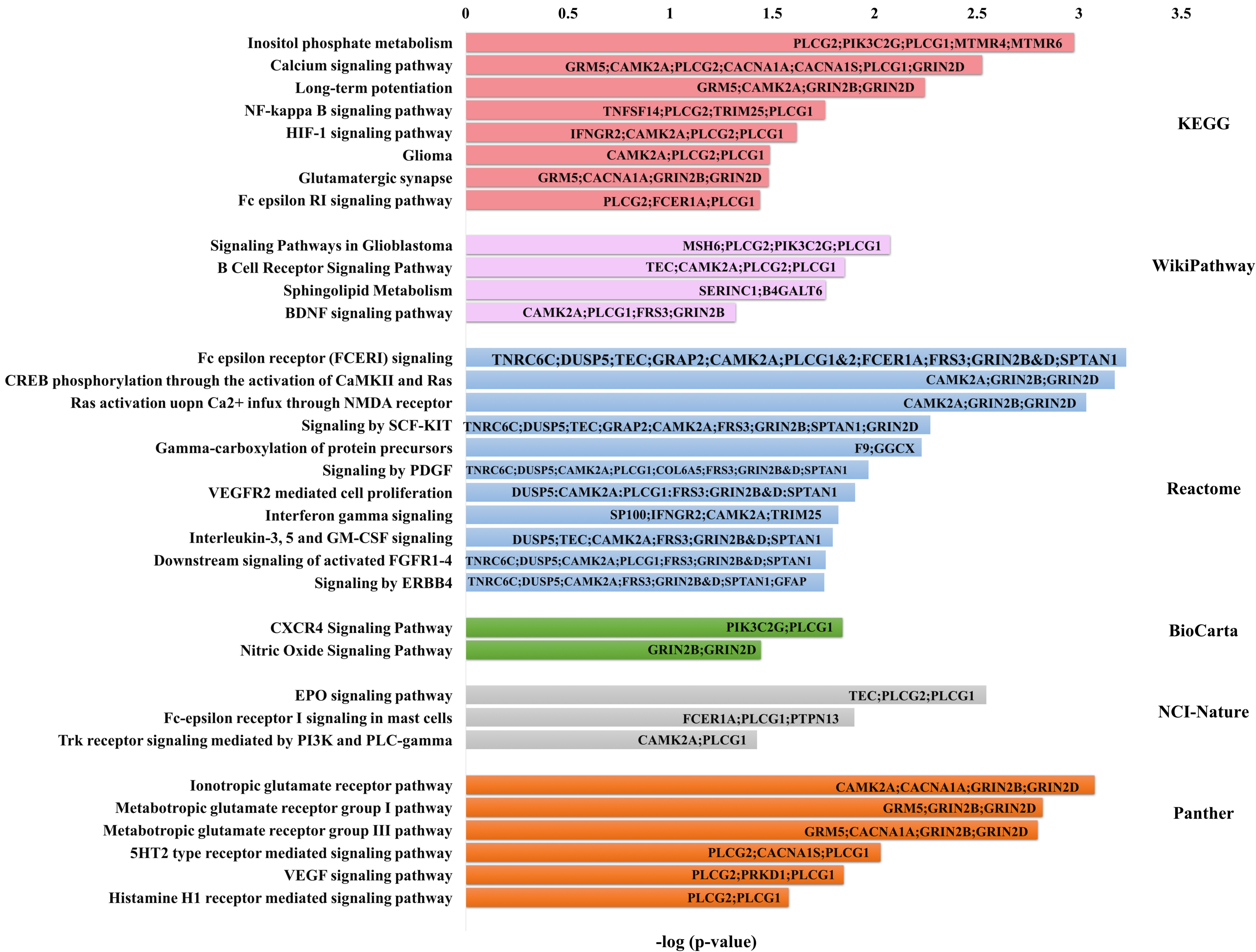

Supplement: S1 Fig — (A) The PR gene signature and (B) PS gene signature have been classified in a set of pathways and ordered according to P-value by mining in the databases, including KEGG (pink), WikiPathways (lilac), Reactome (blue), BioCarta (green), NCI-Nature (grey), and Panther (orange) in Enrichr. (PDF) [file pone.0183969.s002.pdf]

A

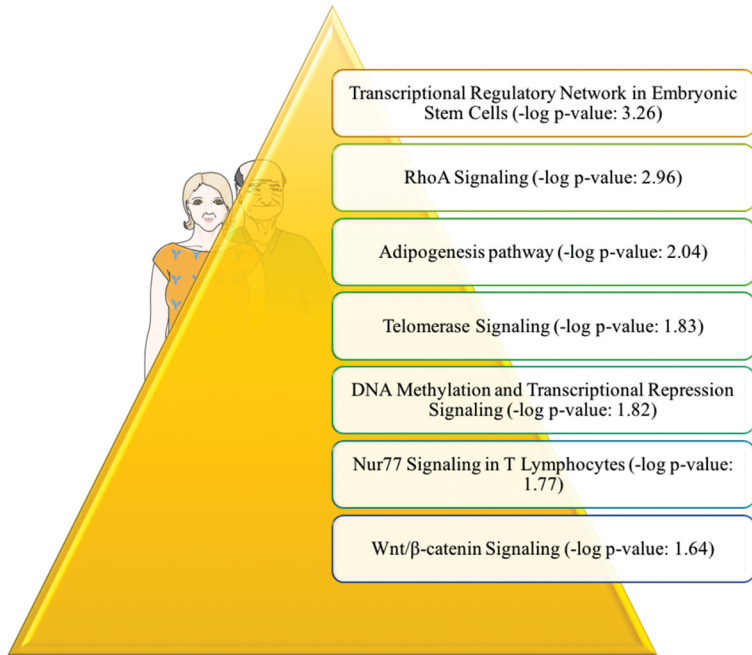

B

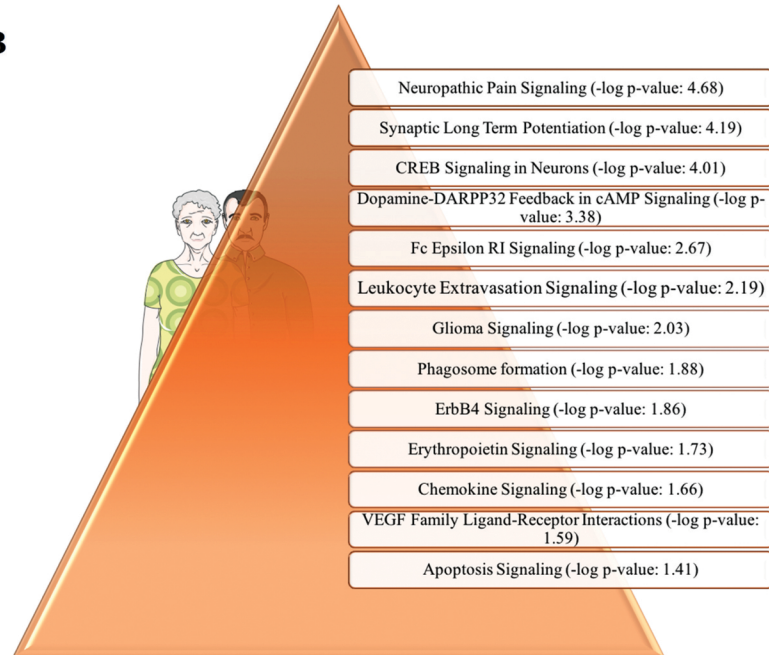

Supplement: S2 Fig — (A) PR-related pathway. (B) PS-related pathway. P < 0.05 is considered statistically significant. (PDF) [file pone.0183969.s003.pdf]
