# Supplementary material for: A systems medicine approach for finding target proteins affecting treatment outcomes in patients with non-Hodgkin lymphoma
Source: PLoS One. 2017 Sep 11;12(9):e0183969. doi: 10.1371/journal.pone.0183969 (PMC5593188; doi:10.1371/journal.pone.0183969)
Supplement: S1 Table — (DOCX) [file pone.0183969.s004.docx]

**S1 Table. Baseline characteristic of NHL patients.**

| **Characteristic** | | **PR NHL patients**  **(10)** | **PS B-cell-NHL**  **(20)** | |
| --- | --- | --- | --- | --- |
| ***Age (years)*** | | | |  |
| Median (range) | | 56 (18-63) | 53 (22-77) | |
| ***Sex*** | | | |  |
| Men (%) | | 6 (67%) | 10 (52%) | |
| Women (%) | | 3 (33%) | 9 (47%) | |
| ***NHL type*** | | | |  |
| Diffuse large-B-cell-lymphoma | | 8 (80%) | 15 (75%) | |
| CD20-positive | ALL CASES | | |  |
| *Other types** | | 2 (20%) | 5 (25%) | |
| ***Primary refractory*** | | - | 5 (25%) | |
| ***Ann Arbor disease stage*** | | | |  |
| I and II | | 5 (50%) | 12 (60%) | |
| III and IV | | 5 (50%) | 8 (40%) | |
| ***Treatment regimen (first line)***** | |  |  | |
| Anthracycline-based regimens (± Rituximab) | | - | 18 (90%) | |
| Other regimens | | - | 2 (10%) | |

* Burkitt’s lymphoma, Mantle-cell lymphoma, Follicular lymphoma, Splenic marginal zone lymphoma and Chronic B-cell lymphoproliferative disorders.

** G-CSF was prescribed for most patients who were under chemotherapy regimen.
