# Supplementary material for: A systems medicine approach for finding target proteins affecting treatment outcomes in patients with non-Hodgkin lymphoma
Source: PLoS One. 2017 Sep 11;12(9):e0183969. doi: 10.1371/journal.pone.0183969 (PMC5593188; doi:10.1371/journal.pone.0183969)
Supplement: S2 Table — (DOCX) [file pone.0183969.s005.docx]

**S2 Table. Baseline characteristic of healthy volunteers.**

| **Characteristic** | | **Control subjects** |
| --- | --- | --- |
| *Number of people enrolled in the study* | | 123 |
| *Number of people selected* | | 92 |
| *Median (range)* | | 40 (11-80) |
| *Men (%)* | | 44% |
| *Women (%)* | | 56% |
| *Participants excluded from the study due to abnormalities in these factors (%)* | RF + ESR + X-ray | 2.5% |
|  | RF+ ESR | 3% |
|  | CRP+ ESR | 1.6% |
|  | RF + X-ray | 0.8% |
|  | ESR+ X-ray | 0.8% |
|  | Chest X-ray | 4% |
|  | ESR | 4% |
|  | Other factors | 8.5% |

**Abbreviations:**

ESR: Erythrocyte sedimentation rate, RF: rheumatoid factor, and CRP: C-reactive protein.
