# Supplementary material for: A systems medicine approach for finding target proteins affecting treatment outcomes in patients with non-Hodgkin lymphoma
Source: PLoS One. 2017 Sep 11;12(9):e0183969. doi: 10.1371/journal.pone.0183969 (PMC5593188; doi:10.1371/journal.pone.0183969)
Supplement: S3 Table — FN7, FT2, and FT4 have similar amino acid sequences (orange). Four peptides FN2 & FN8 (pink) and FT1 & FT9 (blue) were selected for verification by ELISA. (DOCX) [file pone.0183969.s006.docx]

**S3 Table. List of peptides identified through panning on the purified IgG of PR (FNs) and PS (FTs).** FN7, FT2, and FT4 have similar amino acid sequences (orange). Four peptides FN2 & FN8 (pink) and FT1 & FT9 (blue) were selected for verification by ELISA.

| No | Clones  (PR) | Amino acid seq. | Clones  (PS) | Amino acid seq. | |  |
| --- | --- | --- | --- | --- | --- | --- |
| 1 | FN1 | -CKHHHYPHC- | **FT1** | | **-CHRRHGGSC-** | |
| 2 | **FN2** | **-CGSSPNHKC-** | **FT2 and FT4** | | **-CKNSHWWHC-** | |
| 3 | FN3 | -CFPSNMTTC- | FT5 | | -CSRHWWHSC- | |
| 4 | **FN7** | **-CKNSHWWHC-** | FT6 | | -CHTEMKATC- | |
| 5 | **FN8** | **-CRRSDTLYC-** | FT7 | | -CMKGHWRQC- | |
| 6 | FN9 | -CRAHHFHKC- | FT8 | | -CAMASSNGC- | |
| 7 | FN10 | -CMRLAYNTC- | **FT9** | | **-CHFHRPHKC-** | |
| 8 | FN11 | -CPRHHTHKC- | FT11 | | -CKKWFHTHC- | |
