# Supplementary material for: A systems medicine approach for finding target proteins affecting treatment outcomes in patients with non-Hodgkin lymphoma
Source: PLoS One. 2017 Sep 11;12(9):e0183969. doi: 10.1371/journal.pone.0183969 (PMC5593188; doi:10.1371/journal.pone.0183969)
Supplement: S4 Table — The cut-off maximum score for selecting proteins in the Refseq database of BLASTP was considered equal or more than 18.5. Proteins with score more than 18.5 were nominated to investigate in more detail. (A) List of predicted PR proteins, (B) list of predicted PS proteins, and (C) list of proteins which were common between two groups. (DOCX) [file pone.0183969.s007.docx]

**S4 Table. List of proteins predicted from PR- and PS-specific peptides.** The cut-off maximum score for selecting proteins in the Refseq database of BLASTP was considered equal or more than 18.5. Proteins with score more than 18.5 were nominated to investigate in more detail. (A) List of predicted PR proteins, (B) list of predicted PS proteins, and (C) list of proteins which were common between two groups.

| **A.** | |  |  |  |  |  |
| --- | --- | --- | --- | --- | --- | --- |
| *No.* | *Protein* | | | *UniProt accession number* | *Gene ID* | |
| *1* | [C2 domain-containing protein 3 isoform X5](http://blast.ncbi.nlm.nih.gov/Blast.cgi#alnHdr_530396574) | | | Q4AC94 | [C2CD3](http://www.uniprot.org/uniprot/Q4AC94) | |
| *2* | [bone morphogenetic protein 3B isoform X1](http://blast.ncbi.nlm.nih.gov/Blast.cgi#alnHdr_578819253) | | | P12645 | BMP3 | |
| *3* | [immunoglobulin-like domain-containing receptor 1 isoform 3 precursor](http://blast.ncbi.nlm.nih.gov/Blast.cgi#alnHdr_315259080) | | | Q86SU0 | ILDR1 | |
| *4* | [methylcytosine dioxygenase TET2 isoform X5](http://blast.ncbi.nlm.nih.gov/Blast.cgi#alnHdr_578809193) | | | Q6N021 | [TET2](http://www.uniprot.org/uniprot/Q6N021) | |
| *5* | [E3 ubiquitin-protein ligase HECW1 isoform a](http://blast.ncbi.nlm.nih.gov/Blast.cgi#alnHdr_94536811) | | | Q76N89 | HECW1 | |
| *6* | [CDK5 and ABL1 enzyme substrate 1 isoform 3](http://blast.ncbi.nlm.nih.gov/Blast.cgi#alnHdr_374253774) | | | Q8TDN4 | [CABLES1](http://www.uniprot.org/uniprot/Q8TDN4) | |
| *7* | [intersectin-2 isoform 2](http://blast.ncbi.nlm.nih.gov/Blast.cgi#alnHdr_194294525) | | | Q9NZM3 | [ITSN2](http://www.uniprot.org/uniprot/Q9NZM3) | |
| *8* | [titin isoform X4](http://blast.ncbi.nlm.nih.gov/Blast.cgi#alnHdr_530370914) | | | Q8WZ42 | TTN | |
| *9* | [torsin-4A](http://blast.ncbi.nlm.nih.gov/Blast.cgi#alnHdr_116063568) | | | Q9NXH8 | [TOR4A](http://www.uniprot.org/uniprot/Q9NXH8) | |
| *10* | [protein GREB1 isoform X7](http://blast.ncbi.nlm.nih.gov/Blast.cgi#alnHdr_530366992) | | | Q4ZG55 | GREB1 | |
| *11* | [rho GTPase-activating protein 27 isoform c](http://blast.ncbi.nlm.nih.gov/Blast.cgi#alnHdr_226817316) | | | Q6ZUM4 | ARHGAP27 | |
| *12* | [phosphatidylinositol 4-phosphate 3-kinase C2 domain-containing subunit gamma isoform 2](http://blast.ncbi.nlm.nih.gov/Blast.cgi#alnHdr_571026670) | | | O75747 | PIK3C2G | |
| *13* | [FAD synthase isoform 4](http://blast.ncbi.nlm.nih.gov/Blast.cgi#alnHdr_296434312) | | | Q8NFF5 | [FLAD1](http://www.uniprot.org/uniprot/Q8NFF5) | |
| *14* | [HSPB1-associated protein 1](http://blast.ncbi.nlm.nih.gov/Blast.cgi#alnHdr_21314714) | | | Q96EW2 | HSPBAP1 | |
| *15* | [E3 SUMO-protein ligase CBX4](http://blast.ncbi.nlm.nih.gov/Blast.cgi#alnHdr_55770830) | | | O00257 | [CBX4](http://www.google.com/url?sa=t&rct=j&q=&esrc=s&source=web&cd=1&ved=0ahUKEwjbwZG--qXJAhUGYg8KHX7pCtkQFggcMAA&url=http%253A%252F%252Fwww.uniprot.org%252Funiprot%252FO00257&usg=AFQjCNEqg7gC8pVmFPGvqYCRkQPbteYjzA&sig2=CIUrJCY4pBYdCvo67VtShw&bvm=bv.108194040,d.bGQ) | |
| *16* | [equilibrative nucleoside transporter 2 isoform b](http://blast.ncbi.nlm.nih.gov/Blast.cgi#alnHdr_664805964) | | | Q14542 | [SLC29A2](http://www.google.com/url?sa=t&rct=j&q=&esrc=s&source=web&cd=1&ved=0ahUKEwiD4pLw-qXJAhVD2Q4KHa-LAF0QFgghMAA&url=http%253A%252F%252Fwww.uniprot.org%252Funiprot%252FQ14542&usg=AFQjCNH2D7Hu8mls5w2j7mbRmsN6ACGIpA&sig2=SrIMMxuKvib8qIhqYb-mcw&bvm=bv.108194040,d.bGQ) | |
| *17* | [AFG3-like protein 2](http://blast.ncbi.nlm.nih.gov/Blast.cgi#alnHdr_300192933) | | | Q9Y4W6 | AFG3L2 | |
| *18* | [voltage-dependent calcium channel subunit alpha-2/delta-3 isoform X1](http://blast.ncbi.nlm.nih.gov/Blast.cgi#alnHdr_530372845) | | | Q8IZS8 | CACNA2D3 | |
| *19* | [trimeric intracellular cation channel type A](http://blast.ncbi.nlm.nih.gov/Blast.cgi#alnHdr_13129060) | | | Q9H6F2 | TMEM38A | |
| *20* | [protein naked cuticle homolog 1 isoform X1](http://blast.ncbi.nlm.nih.gov/Blast.cgi#alnHdr_530424451) | | | Q969G9 | NKD1 | |
| *21* | [ERC protein 2](http://blast.ncbi.nlm.nih.gov/Blast.cgi#alnHdr_34577114) | | | O15083 | ERC2 | |
| *22* | [keratin, type II cuticular Hb2](http://blast.ncbi.nlm.nih.gov/Blast.cgi#alnHdr_27477127) | | | Q9NSB4 | KRT82 | |
| *23* | [putative RNA-binding protein 15 isoform 2](http://blast.ncbi.nlm.nih.gov/Blast.cgi#alnHdr_319996623) | | | Q96T37 | RBM15 | |
| *24* | [uncharacterized protein C8orf74](http://blast.ncbi.nlm.nih.gov/Blast.cgi#alnHdr_91176323) | | | Q6P047 | C8ORF74 | |
| *25* | [constitutive coactivator of PPAR-gamma-like protein 2 isoform 2](http://blast.ncbi.nlm.nih.gov/Blast.cgi#alnHdr_597709752) | | | Q9NX05 | FAM120C | |
| *26* | [DNA polymerase alpha catalytic subunit isoform X2](http://blast.ncbi.nlm.nih.gov/Blast.cgi#alnHdr_578837922) | | | P09884 | POLA1 | |
| *27* | [basement membrane-specific heparan sulfate proteoglycan core protein isoform X10](http://blast.ncbi.nlm.nih.gov/Blast.cgi#alnHdr_578799003) | | | P98160 | HSPG2 | |
| *28* | [ADP-ribosylation factor GTPase-activating protein 2 isoform 2](http://blast.ncbi.nlm.nih.gov/Blast.cgi#alnHdr_338827663) | | | Q8N6H7 | ARFGAP2 | |
| *29* | [U11/U12 small nuclear ribonucleoprotein 48 kDa protein](http://blast.ncbi.nlm.nih.gov/Blast.cgi#alnHdr_71143123) | | | Q6IEG0 | SNRNP48 | |
| *30* | [gonadotropin-releasing hormone receptor isoform 1](http://blast.ncbi.nlm.nih.gov/Blast.cgi#alnHdr_4504059) | | | P30968 | GNRHR | |
| *31* | [histidine-rich carboxyl terminus protein 1](http://blast.ncbi.nlm.nih.gov/Blast.cgi#alnHdr_89886261) | | | Q6UXD1 | HRCT1 | |
| *32* | [heart- and neural crest derivatives-expressed protein 1 isoform X1](http://blast.ncbi.nlm.nih.gov/Blast.cgi#alnHdr_530380724) | | | O96004 | HAND1 | |
| *33* | [cbp/p300-interacting transactivator 2 isoform 1](http://blast.ncbi.nlm.nih.gov/Blast.cgi#alnHdr_19923343) | | | Q99967 | CITED2 | |
| *34* | [homeobox protein OTX1 isoform X1](http://blast.ncbi.nlm.nih.gov/Blast.cgi#alnHdr_578802923) | | | P32242 | OTX1 | |
| *35* | [transcription factor MafB](http://blast.ncbi.nlm.nih.gov/Blast.cgi#alnHdr_23308699) | | | Q9Y5Q3 | MAFB | |
| *36* | [cyclic AMP-responsive element-binding protein 5 isoform delta](http://blast.ncbi.nlm.nih.gov/Blast.cgi#alnHdr_59938776) | | | Q02930 | CREB5 | |
| *37* | [forkhead box protein B2](http://blast.ncbi.nlm.nih.gov/Blast.cgi#alnHdr_61966923) | | | Q5VYV0 | FOXB2 | |
| *38* | [hepatocyte nuclear factor 6](http://blast.ncbi.nlm.nih.gov/Blast.cgi#alnHdr_24307887) | | | Q9UBC0 | ONECUT1 | |
| *39* | [small conductance calcium-activated potassium channel protein 2 isoform X1](http://blast.ncbi.nlm.nih.gov/Blast.cgi#alnHdr_578810350) | | | Q9H2S1 | KCNN2 | |
| *40* | [SKI family transcriptional corepressor 2 isoform 1](http://blast.ncbi.nlm.nih.gov/Blast.cgi#alnHdr_491228692) | | | Q2VWA4 | SKOR2 | |
| *41* | [probable helicase with zinc finger domain isoform X6](http://blast.ncbi.nlm.nih.gov/Blast.cgi#alnHdr_578831752) | | | P42694 | HELZ | |
| *42* | [chromodomain-helicase-DNA-binding protein 8 isoform 2](http://blast.ncbi.nlm.nih.gov/Blast.cgi#alnHdr_114326455) | | | Q9HCK8 | CHD8 | |
| *43* | [ephexin-1 isoform 2](http://blast.ncbi.nlm.nih.gov/Blast.cgi#alnHdr_166197690) | | | Q8N5V2 | NGEF | |
| *44* | [NHS-like protein 2](http://blast.ncbi.nlm.nih.gov/Blast.cgi#alnHdr_254692833) | | | Q5HYW2 | NHSL2 | |
| *45* | [E3 ubiquitin-protein ligase RNF213 isoform 3](http://blast.ncbi.nlm.nih.gov/Blast.cgi#alnHdr_597709777) | | | Q63HN8 | RNF213 | |
| *46* | [tripartite motif-containing protein 77 isoform 2](http://blast.ncbi.nlm.nih.gov/Blast.cgi#alnHdr_429836863) | | | I1YAP6 | TRIM77 | |
| *47* | [centrosomal protein of 72 kDa isoform X3](http://blast.ncbi.nlm.nih.gov/Blast.cgi#alnHdr_530378855) | | | Q9P209 | CEP72 | |
| *48* | [probable ATP-dependent RNA helicase DHX58](http://blast.ncbi.nlm.nih.gov/Blast.cgi#alnHdr_149408122) | | | Q96C10 | DHX58 | |
| *49* | [transmembrane protein 244](http://blast.ncbi.nlm.nih.gov/Blast.cgi#alnHdr_58197570) | | | Q5VVB8 | TMEM244 | |
| *50* | [NUAK family SNF1-like kinase 2 isoform X1](http://blast.ncbi.nlm.nih.gov/Blast.cgi#alnHdr_530365437) | | | Q9H093 | NUAK2 | |
| *51* | [E3 ubiquitin-protein ligase MYCBP2](http://blast.ncbi.nlm.nih.gov/Blast.cgi#alnHdr_291190787) | | | O75592 | MYCBP2 | |
| *52* | [mediator of RNA polymerase II transcription subunit 13-like isoform X3](http://blast.ncbi.nlm.nih.gov/Blast.cgi#alnHdr_578823481) | | | Q71F56 | MED13L | |
| *53* | [heat shock transcription factor, X-linked](http://blast.ncbi.nlm.nih.gov/Blast.cgi#alnHdr_7706603) | | | Q9UBD0 | HSFX1 | |
| *54* | [histone deacetylase 1](http://blast.ncbi.nlm.nih.gov/Blast.cgi#alnHdr_13128860) | | | Q13547 | HDAC1 | |
| *55* | [histone deacetylase 2](http://blast.ncbi.nlm.nih.gov/Blast.cgi#alnHdr_293336691) | | | Q92769 | HDAC2 | |
| *56* | [60S ribosomal protein L34](http://blast.ncbi.nlm.nih.gov/Blast.cgi#alnHdr_16117787) | | | P49207 | RPL34 | |
| *57* | [IQ domain-containing protein F6](http://blast.ncbi.nlm.nih.gov/Blast.cgi#alnHdr_254028213) | | | A8MYZ5 | IQCF6 | |
| *58* | [glutamate receptor ionotropic, delta-1 isoform X1](http://blast.ncbi.nlm.nih.gov/Blast.cgi#alnHdr_578819344) | | | Q9ULK0 | [GRID1](http://www.uniprot.org/uniprot/Q9ULK0) | |
| *59* | [purine-rich element-binding protein gamma isoform B](http://blast.ncbi.nlm.nih.gov/Blast.cgi#alnHdr_62865862) | | | Q9UJV8 | PURG | |
| *60* | [paired immunoglobulin-like type 2 receptor alpha isoform 1 precursor](http://blast.ncbi.nlm.nih.gov/Blast.cgi#alnHdr_30179909) | | | Q9UKJ1 | PILRA | |
| *61* | [transcriptional regulator ERG isoform 6](http://blast.ncbi.nlm.nih.gov/Blast.cgi#alnHdr_343478184) | | | P11308 | ERG | |
| *62* | [coiled-coil domain-containing protein 183](http://blast.ncbi.nlm.nih.gov/Blast.cgi#alnHdr_289547545) | | | Q5T5S1 | CCDC183 | |
| *63* | [chloride intracellular channel protein 6](http://blast.ncbi.nlm.nih.gov/Blast.cgi#alnHdr_27894378) | | | Q96NY7 | CLIC6 | |
| *64* | [X6](http://blast.ncbi.nlm.nih.gov/Blast.cgi#alnHdr_530383351) | | | Q96NJ5 | KLHL32 | |
| *65* | [AP-5 complex subunit zeta-1](http://blast.ncbi.nlm.nih.gov/Blast.cgi#alnHdr_82546847) | | | O43299 | AP5Z1 | |
| *66* | [glycine receptor subunit alpha-1 isoform X2](http://blast.ncbi.nlm.nih.gov/Blast.cgi#alnHdr_530380480) | | | P23415 | GLRA1 | |
| *67* | [calcium/calmodulin-dependent protein kinase kinase 1 isoform X3](http://blast.ncbi.nlm.nih.gov/Blast.cgi#alnHdr_530410688) | | | Q8N5S9 | CAMKK1 | |
| *68* | [serine/threonine-protein kinase TBK1](http://blast.ncbi.nlm.nih.gov/Blast.cgi#alnHdr_7019547) | | | Q9UHD2 | TBK1 | |
| *69* | [signal-induced proliferation-associated 1-like protein 1 isoform 3](http://blast.ncbi.nlm.nih.gov/Blast.cgi#alnHdr_545746375) | | | O43166 | SIPA1L1 | |
| *70* | [kazrin isoform C](http://blast.ncbi.nlm.nih.gov/Blast.cgi#alnHdr_63999741) | | | Q674X7 | KAZN | |
| *71* | [solute carrier family 28 member 3](http://blast.ncbi.nlm.nih.gov/Blast.cgi#alnHdr_11545853) | | | Q9HAS3 | SLC28A3 | |
| *72* | [lysosomal alpha-mannosidase isoform 2 precursor](http://blast.ncbi.nlm.nih.gov/Blast.cgi#alnHdr_291045220) | | | O00754 | [MAN2B1](http://www.google.com/url?sa=t&rct=j&q=&esrc=s&source=web&cd=1&ved=0ahUKEwjUyePg_JXLAhXDCJoKHYCxA5AQFgghMAA&url=http%253A%252F%252Fwww.uniprot.org%252Funiprot%252FO00754&usg=AFQjCNH1W7E8r2fup0bEQtj6Y2lnZR3ebg&sig2=MMKqYz8RtHI69n3ZR94UkA&bvm=bv.115339255,d.bGs&cad=rja) | |
| *73* | [39S ribosomal protein L49, mitochondrial](http://blast.ncbi.nlm.nih.gov/Blast.cgi#alnHdr_4826649) | | | Q13405 | MRPL49 | |
| *74* | [matrix-remodeling-associated protein 8 isoform 3 precursor](http://blast.ncbi.nlm.nih.gov/Blast.cgi#alnHdr_543423803) | | | Q9BRK3 | MXRA8 | |
| *75* | [DDB1- and CUL4-associated factor 13](http://blast.ncbi.nlm.nih.gov/Blast.cgi#alnHdr_229892270) | | | Q9NV06 | DCAF13 | |
| *76* | [axin-2 isoform X4](http://blast.ncbi.nlm.nih.gov/Blast.cgi#alnHdr_530412957) | | | Q9Y2T1 | AXIN2 | |
| *77* | [E3 ubiquitin-protein ligase Arkadia isoform 3](http://blast.ncbi.nlm.nih.gov/Blast.cgi#alnHdr_395455083) | | | Q6ZNA4 | RNF111 | |
| *78* | [engulfment and cell motility protein 1 isoform 2](http://blast.ncbi.nlm.nih.gov/Blast.cgi#alnHdr_18765702) | | | Q92556 | ELMO1 | |
| *79* | [N-terminal EF-hand calcium-binding protein 2 isoform X1](http://blast.ncbi.nlm.nih.gov/Blast.cgi#alnHdr_578829003) | | | Q7Z6G3 | NECAB2 | |
| *80* | [engulfment and cell motility protein 3](http://blast.ncbi.nlm.nih.gov/Blast.cgi#alnHdr_87298935) | | | Q96BJ8 | ELMO3 | |
| *81* | [DNA topoisomerase 2-alpha](http://blast.ncbi.nlm.nih.gov/Blast.cgi#alnHdr_19913406) | | | P11388 | TOP2A | |
| *82* | [immunoglobulin superfamily containing leucine-rich repeat protein precursor](http://blast.ncbi.nlm.nih.gov/Blast.cgi#alnHdr_5031809) | | | O14498 | ISLR | |
| *83* | [uncharacterized protein C7orf62](http://blast.ncbi.nlm.nih.gov/Blast.cgi#alnHdr_22749409) | | | Q8TBZ9 | C7orf62 | |
| *84* | [CUGBP Elav-like family member 6 isoform 3](http://blast.ncbi.nlm.nih.gov/Blast.cgi#alnHdr_289547743) | | | Q96J87 | CELF6 | |
| *85* | [P2Y purinoceptor 4](http://blast.ncbi.nlm.nih.gov/Blast.cgi#alnHdr_4505561) | | | P51582 | P2RY4 | |
| *86* | [telomeric repeat-binding factor 2 isoform X4](http://blast.ncbi.nlm.nih.gov/Blast.cgi#alnHdr_530424267) | | | Q15554 | TERF2 | |
| *87* | [interleukin-6 receptor subunit beta isoform 3 precursor](http://blast.ncbi.nlm.nih.gov/Blast.cgi#alnHdr_300244535) | | | P40189 | IL6ST | |
| *88* | [transient receptor potential cation channel subfamily M member 8](http://blast.ncbi.nlm.nih.gov/Blast.cgi#alnHdr_109689695) | | | Q7Z2W7 | TRPM8 | |
| *89* | [ankyrin repeat domain-containing protein 26 isoform X8](http://blast.ncbi.nlm.nih.gov/Blast.cgi#alnHdr_578818370) | | | Q9UPS8 | ANKRD26 | |
| *90* | [sucrase-isomaltase, intestinal](http://blast.ncbi.nlm.nih.gov/Blast.cgi#alnHdr_157364974) | | | P14410 | SI | |
| *91* | [protein furry homolog-like isoform X12](http://blast.ncbi.nlm.nih.gov/Blast.cgi#alnHdr_530376644) | | | O94915 | FRYL | |
| *92* | [histone-lysine N-methyltransferase EHMT2 isoform b](http://blast.ncbi.nlm.nih.gov/Blast.cgi#alnHdr_156142199) | | | Q96KQ7 | EHMT2 | |
| *93* | [nuclear receptor coactivator 7 isoform 4](http://blast.ncbi.nlm.nih.gov/Blast.cgi#alnHdr_313850981) | | | Q8NI08 | NCOA7 | |
| *94* | [Friend leukemia integration 1 transcription factor isoform 4](http://blast.ncbi.nlm.nih.gov/Blast.cgi#alnHdr_401871078) | | | Q01543 | FLI1 | |
| *95* | [40S ribosomal protein S4, Y isoform 2](http://blast.ncbi.nlm.nih.gov/Blast.cgi#alnHdr_88703062) | | | Q8TD47 | RPS4Y2 | |
| *96* | [protein EVI2B precursor](http://blast.ncbi.nlm.nih.gov/Blast.cgi#alnHdr_148227044) | | | P34910 | EVI2B | |
| *97* | [Abelson tyrosine-protein kinase 2 isoform e](http://blast.ncbi.nlm.nih.gov/Blast.cgi#alnHdr_209862778) | | | P42684 | ABL2 | |
| *98* | [epithelial splicing regulatory protein 1 isoform 3](http://blast.ncbi.nlm.nih.gov/Blast.cgi#alnHdr_170763529) | | | Q6NXG1 | ESRP1 | |
| *99* | [WD repeat-containing protein 44 isoform 3](http://blast.ncbi.nlm.nih.gov/Blast.cgi#alnHdr_296841075) | | | Q5JSH3 | WDR44 | |
| *100* | [cyclin-T2 isoform X3](http://blast.ncbi.nlm.nih.gov/Blast.cgi#alnHdr_530369531) | | | O60583 | CCNT2 | |
| *101* | [heat shock factor protein 5](http://blast.ncbi.nlm.nih.gov/Blast.cgi#alnHdr_612407780) | | | Q4G112 | HSF5 | |
| *102* | [prolyl 4-hydroxylase subunit alpha-2 isoform 2 precursor](http://blast.ncbi.nlm.nih.gov/Blast.cgi#alnHdr_63252891) | | | O15460 | P4HA2 | |
| *103* | [sprT-like domain-containing protein Spartan isoform c](http://blast.ncbi.nlm.nih.gov/Blast.cgi#alnHdr_387762597) | | | Q9H040 | SPRTN | |
| *104* | [proline-rich protein 29 isoform 3](http://blast.ncbi.nlm.nih.gov/Blast.cgi#alnHdr_300388158) | | | P0C7W0 | PRR29 | |
| *105* | [androgen-induced gene 1 protein isoform b](http://blast.ncbi.nlm.nih.gov/Blast.cgi#alnHdr_557440814) | | | Q9NVV5 | AIG1 | |
| *106* | [proline-rich transmembrane protein 1](http://blast.ncbi.nlm.nih.gov/Blast.cgi#alnHdr_77681565) | | | Q99946 | PRRT1 | |
| *107* | [homeobox protein Meis1](http://blast.ncbi.nlm.nih.gov/Blast.cgi#alnHdr_4505151) | | | O00470 | MEIS1 | |
| *108* | [forkhead box protein F2](http://blast.ncbi.nlm.nih.gov/Blast.cgi#alnHdr_4557595) | | | Q12947 | FOXF2 | |
| *109* | [serine/threonine-protein kinase NLK isoform X1](http://blast.ncbi.nlm.nih.gov/Blast.cgi#alnHdr_530411054) | | | Q9UBE8 | NLK | |
| *110* | [potassium voltage-gated channel subfamily G member 4](http://blast.ncbi.nlm.nih.gov/Blast.cgi#alnHdr_27436996) | | | Q8TDN1 | KCNG4 | |
| *111* | [GMP synthase [glutamine-hydrolyzing]](http://blast.ncbi.nlm.nih.gov/Blast.cgi#alnHdr_4504035) | | | P49915 | GMPS | |
| *112* | [nuclear fragile X mental retardation-interacting protein 2](http://blast.ncbi.nlm.nih.gov/Blast.cgi#alnHdr_32698730) | | | Q7Z417 | NUFIP2 | |
| *113* | [E3 ubiquitin-protein ligase RNF43 precursor](http://blast.ncbi.nlm.nih.gov/Blast.cgi#alnHdr_56711322) | | | Q68DV7 | RNF43 | |
| *114* | [cAMP-specific 3',5'-cyclic phosphodiesterase 4D isoform PDE4D4](http://blast.ncbi.nlm.nih.gov/Blast.cgi#alnHdr_157277988) | | | Q08499 | PDE4D | |
| *115* | [zinc finger protein 281 isoform 2](http://blast.ncbi.nlm.nih.gov/Blast.cgi#alnHdr_526253066) | | | Q9Y2X9 | ZNF281 | |
| *116* | [transient receptor potential cation channel subfamily M member 6 isoform X4](http://blast.ncbi.nlm.nih.gov/Blast.cgi#alnHdr_578817065) | | | Q9BX84 | TRPM6 | |
| *117* | [carboxypeptidase D isoform 2](http://blast.ncbi.nlm.nih.gov/Blast.cgi#alnHdr_315138990) | | | O75976 | CPD | |
| *118* | [serine/threonine-protein kinase Nek1 isoform X2](http://blast.ncbi.nlm.nih.gov/Blast.cgi#alnHdr_578809149) | | | Q96PY6 | NEK1 | |
| *119* | [upstream-binding protein 1 isoform X1](http://blast.ncbi.nlm.nih.gov/Blast.cgi#alnHdr_578806543) | | | Q9NZI7 | UBP1 | |
| *120* | [histone demethylase UTY isoform 1](http://blast.ncbi.nlm.nih.gov/Blast.cgi#alnHdr_33188429) | | | O14607 | UTY | |
| *121* | [formin-2](http://blast.ncbi.nlm.nih.gov/Blast.cgi#alnHdr_160707881) | | | Q9NZ56 | FMN2 | |
| *122* | [kinase D-interacting substrate of 220 kDa](http://blast.ncbi.nlm.nih.gov/Blast.cgi#alnHdr_55741641) | | | Q9ULH0 | KIDINS220 | |
| *123* | [T-lymphocyte activation antigen CD86 isoform 4 precursor](http://blast.ncbi.nlm.nih.gov/Blast.cgi#alnHdr_332634950) | | | P42081 | CD86 | |
| *124* | [helicase ARIP4](http://blast.ncbi.nlm.nih.gov/Blast.cgi#alnHdr_168823443) | | | Q9Y4B4 | RAD54L2 | |
| *125* | [telomerase protein component 1](http://blast.ncbi.nlm.nih.gov/Blast.cgi#alnHdr_21536371) | | | Q99973 | TEP1 | |
| *126* | [serine/arginine-rich splicing factor 12 isoform X1](http://blast.ncbi.nlm.nih.gov/Blast.cgi#alnHdr_578812424) | | | Q8WXF0 | SRSF12 | |
| *127* | [cathepsin K preproprotein](http://blast.ncbi.nlm.nih.gov/Blast.cgi#alnHdr_4503151) | | | P43235 | CTSK | |
| *128* | [cdc42 effector protein 1](http://blast.ncbi.nlm.nih.gov/Blast.cgi#alnHdr_23238226) | | | Q00587 | CDC42EP1 | |
| *129* | [gasdermin-C](http://blast.ncbi.nlm.nih.gov/Blast.cgi#alnHdr_13899221) | | | Q9BYG8 | GSDMC | |
| *130* | [serine/threonine-protein phosphatase with EF-hands 1 isoform 3](http://blast.ncbi.nlm.nih.gov/Blast.cgi#alnHdr_23312378) | | | O14829 | PPEF1 | |
| *131* | [lactotransferrin isoform 2](http://blast.ncbi.nlm.nih.gov/Blast.cgi#alnHdr_312433998) | | | P02788 | LTF | |
| *132* | [B-cell lymphoma/leukemia 11A isoform 2](http://blast.ncbi.nlm.nih.gov/Blast.cgi#alnHdr_20336307) | | | Q9H165 | BCL11A | |
| *133* | [B-cell lymphoma/leukemia 11B isoform 4](http://blast.ncbi.nlm.nih.gov/Blast.cgi#alnHdr_532691789) | | | Q9C0K0 | BCL11B | |
| *134* | [NACHT, LRR and PYD domains-containing protein 6 isoform 2](http://blast.ncbi.nlm.nih.gov/Blast.cgi#alnHdr_452085173) | | | P59044 | NLRP6 | |
| *135* | [citron Rho-interacting kinase isoform X3](http://blast.ncbi.nlm.nih.gov/Blast.cgi#alnHdr_530401185) | | | O14578 | CIT | |
| *136* | [myotubularin-related protein 13 isoform X2](http://blast.ncbi.nlm.nih.gov/Blast.cgi#alnHdr_530395847) | | | Q86WG5 | SBF2 | |
| *137* | [cytoplasmic dynein 2 heavy chain 1 isoform X1](http://blast.ncbi.nlm.nih.gov/Blast.cgi#alnHdr_578822370) | | | Q8NCM8 | DYNC2H1 | |
| *138* | [BMP-binding endothelial regulator protein isoform X3](http://blast.ncbi.nlm.nih.gov/Blast.cgi#alnHdr_530384519) | | | Q8N8U9 | BMPER | |
| *139* | [SEC14-like protein 5](http://blast.ncbi.nlm.nih.gov/Blast.cgi#alnHdr_150010661) | | | O43304 | SEC14L5 | |
| *140* | [MAP7 domain-containing protein 2 isoform 1](http://blast.ncbi.nlm.nih.gov/Blast.cgi#alnHdr_270483742) | | | Q96T17 | MAP7D2 | |
| *141* | [coiled-coil domain-containing protein 85A isoform X4](http://blast.ncbi.nlm.nih.gov/Blast.cgi#alnHdr_530367198) | | | Q96PX6 | CCDC85A | |
| *142* | [protein piccolo isoform 2](http://blast.ncbi.nlm.nih.gov/Blast.cgi#alnHdr_150170670) | | | Q9Y6V0 | PCLO | |
| *143* | [ecto-ADP-ribosyltransferase 4 precursor](http://blast.ncbi.nlm.nih.gov/Blast.cgi#alnHdr_61835134) | | | Q93070 | ART4 | |
| *144* | [T-cell surface protein tactile isoform X8](http://blast.ncbi.nlm.nih.gov/Blast.cgi#alnHdr_578806981) | | | P40200 | CD96 | |
| *145* | [solute carrier organic anion transporter family member 6A1 isoform 2](http://blast.ncbi.nlm.nih.gov/Blast.cgi#alnHdr_573014755) | | | Q86UG4 | SLCO6A1 | |
| *146* | [mucin-3A](http://blast.ncbi.nlm.nih.gov/Blast.cgi#alnHdr_578795852) | | | Q02505 | MUC3A | |
| *147* | [putative sodium-coupled neutral amino acid transporter 10 isoform b](http://blast.ncbi.nlm.nih.gov/Blast.cgi#alnHdr_20070376) | | | Q9HBR0 | SLC38A10 | |
| *148* | [DNA damage-induced apoptosis suppressor protein](http://blast.ncbi.nlm.nih.gov/Blast.cgi#alnHdr_194239694) | | | Q8IXT1 | DDIAS | |
| *149* | [Usher syndrome type-1G protein isoform 2](http://blast.ncbi.nlm.nih.gov/Blast.cgi#alnHdr_542133068) | | | Q495M9 | USH1G | |
| *150* | [cis-aconitate decarboxylase](http://blast.ncbi.nlm.nih.gov/Blast.cgi#alnHdr_385719224) | | | A6NK06 | IRG1 | |
| *151* | [zinc finger protein 630 isoform 3](http://blast.ncbi.nlm.nih.gov/Blast.cgi#alnHdr_532524969) | | | Q2M218 | ZNF630 | |
| *152* | [probable inactive 1-aminocyclopropane-1-carboxylate synthase-like protein 2](http://blast.ncbi.nlm.nih.gov/Blast.cgi#alnHdr_149944424) | | | Q4AC99 | ACCSL | |
| *153* | [sentrin-specific protease 2](http://blast.ncbi.nlm.nih.gov/Blast.cgi#alnHdr_54607091) | | | Q9HC62 | SENP2 | |
| *154* | [cyclic AMP-dependent transcription factor ATF-6 alpha isoform X2](http://blast.ncbi.nlm.nih.gov/Blast.cgi#alnHdr_578800689) | | | P18850 | ATF6S | |
| *155* | [leucine-rich repeat and calponin homology domain-containing protein 4 isoform 2](http://blast.ncbi.nlm.nih.gov/Blast.cgi#alnHdr_584277014) | | | O75427 | LRCH4 | |
| *156* | [phosphatidate phosphatase LPIN1 isoform 1](http://blast.ncbi.nlm.nih.gov/Blast.cgi#alnHdr_22027648) | | | Q14693 | LPIN1 | |
| *157* | [hemojuvelin isoform b](http://blast.ncbi.nlm.nih.gov/Blast.cgi#alnHdr_44662823) | | | Q6ZVN8 | HFE2 | |
| *158* | [homeobox protein Nkx-2.3](http://blast.ncbi.nlm.nih.gov/Blast.cgi#alnHdr_148746211) | | | Q8TAU0 | NKX2-3 | |
| *159* | [RING finger and transmembrane domain-containing protein 2 isoform 2 .](http://blast.ncbi.nlm.nih.gov/Blast.cgi#alnHdr_158186612) | | | Q96EX2 | RNFT2 | |
| *160* | [neurexin 3 isoform 2 precursor](http://blast.ncbi.nlm.nih.gov/Blast.cgi#alnHdr_41350305) | | | Q9Y4C0 | NRXN3 | |
| *161* | [neurexin-1-beta isoform X14](http://blast.ncbi.nlm.nih.gov/Blast.cgi#alnHdr_578803284) | | | P58400 | NRXN1 | |
| *162* | [protein naked cuticle homolog 2 isoform 1](http://blast.ncbi.nlm.nih.gov/Blast.cgi#alnHdr_14916427) | | | Q969F2 | NKD2 | |
| *163* | [rap guanine nucleotide exchange factor 3 isoform X4](http://blast.ncbi.nlm.nih.gov/Blast.cgi#alnHdr_530399604) | | | O95398 | RAPGEF3 | |
| *164* | [sorting nexin-18 isoform a](http://blast.ncbi.nlm.nih.gov/Blast.cgi#alnHdr_157057545) | | | Q96RF0 | SNX18 | |
| *165* | [regulation of nuclear pre-mRNA domain-containing protein 2 isoform X3](http://blast.ncbi.nlm.nih.gov/Blast.cgi#alnHdr_530364450) | | | Q5VT52 | RPRD2 | |
| *166* | [zinc transporter ZIP10 precursor](http://blast.ncbi.nlm.nih.gov/Blast.cgi#alnHdr_55741750) | | | Q9ULF5 | SLC39A10 | |
| *167* | [periodic tryptophan protein 2 homolog](http://blast.ncbi.nlm.nih.gov/Blast.cgi#alnHdr_578797770) | | | Q15269 | PWP2 | |
| *168* | [beta-1,4-N-acetylgalactosaminyltransferase 3](http://blast.ncbi.nlm.nih.gov/Blast.cgi#alnHdr_71043500) | | | E9PHD9 | B4GALNT3 | |
| *169* | [POM121-like protein 2](http://blast.ncbi.nlm.nih.gov/Blast.cgi#alnHdr_253683544) | | | Q96KW2 | POM121L2 | |
| *170* | [contactin-associated protein-like 4 isoform 2](http://blast.ncbi.nlm.nih.gov/Blast.cgi#alnHdr_148664242) | | | Q9C0A0 | CNTNAP4 | |
| *171* | [neogenin isoform 3 precursor](http://blast.ncbi.nlm.nih.gov/Blast.cgi#alnHdr_290655729) | | | Q92859 | NEO1 | |
| *172* | [tetratricopeptide repeat protein 19, mitochondrial isoform 2](http://blast.ncbi.nlm.nih.gov/Blast.cgi#alnHdr_406601116) | | | Q6DKK2 | TTC19 | |
| *173* | [protein shisa-7 precursor](http://blast.ncbi.nlm.nih.gov/Blast.cgi#alnHdr_223633890) | | | A6NL88 | SHISA7 | |
| *174* | [frizzled-6 isoform b](http://blast.ncbi.nlm.nih.gov/Blast.cgi#alnHdr_257471001) | | | O60353 | FZD6 | |
| *175* | [zona pellucida sperm-binding protein 2 isoform X1](http://blast.ncbi.nlm.nih.gov/Blast.cgi#alnHdr_530409068) | | | Q05996 | ZP2 | |
| *176* | [cytoskeleton-associated protein 5 isoform a](http://blast.ncbi.nlm.nih.gov/Blast.cgi#alnHdr_57164942) | | | Q14008 | CKAP5 | |
| *177* | [multiple myeloma tumor-associated protein 2](http://blast.ncbi.nlm.nih.gov/Blast.cgi#alnHdr_13236559) | | | Q9BU76 | MMTAG2 | |
| *178* | [mitochondrial fission regulator 2](http://blast.ncbi.nlm.nih.gov/Blast.cgi#alnHdr_39653315) | | | Q6P444 | MTFR2 | |
| *179* | [serine incorporator 1 precursor](http://blast.ncbi.nlm.nih.gov/Blast.cgi#alnHdr_24308213) | | | Q9NRX5 | SERINC1 | |
| *180* | [myotubularin-related protein 6 isoform X1](http://blast.ncbi.nlm.nih.gov/Blast.cgi#alnHdr_578825077) | | | Q9Y217 | MTMR6 | |
| *181* | [retinoic acid receptor gamma isoform X1](http://blast.ncbi.nlm.nih.gov/Blast.cgi#alnHdr_530400634) | | | P13631 | RARG | |
| *182* | [tyrosine-protein kinase Tec isoform X1](http://blast.ncbi.nlm.nih.gov/Blast.cgi#alnHdr_530376704) | | | P42680 | TEC | |
| *183* | [ankyrin repeat domain-containing protein 6 isoform X6](http://blast.ncbi.nlm.nih.gov/Blast.cgi#alnHdr_530383069) | | | Q9Y2G4 | ANKRD6 | |
| *184* | [rho GTPase-activating protein 9 isoform 2](http://blast.ncbi.nlm.nih.gov/Blast.cgi#alnHdr_122939151) | | | Q9BRR9 | ARHGAP9 | |
| *185* | [scavenger receptor cysteine-rich type 1 protein M130 isoform b precursor](http://blast.ncbi.nlm.nih.gov/Blast.cgi#alnHdr_344179112) | | | Q86VB7 | CD163 | |
| *186* | [brefeldin A-inhibited guanine nucleotide-exchange protein 2 isoform X2](http://blast.ncbi.nlm.nih.gov/Blast.cgi#alnHdr_578835710) | | | Q9Y6D5 | ARFGEF2 | |
| *187* | [tectonin beta-propeller repeat-containing protein 2 isoform 2](http://blast.ncbi.nlm.nih.gov/Blast.cgi#alnHdr_289547517) | | | O15040 | TECPR2 | |
| *188* | [spermatogenesis-associated protein 31E1](http://blast.ncbi.nlm.nih.gov/Blast.cgi#alnHdr_155029550) | | | Q6ZUB1 | SPATA31E1 |  |
| *189* | [E3 ubiquitin-protein ligase SHPRH isoform X8](http://blast.ncbi.nlm.nih.gov/Blast.cgi#alnHdr_578812645) | | | Q149N8 | SHPRH | |
| *190* | [brefeldin A-inhibited guanine nucleotide-exchange protein 1 isoform X4](http://blast.ncbi.nlm.nih.gov/Blast.cgi#alnHdr_578815582) | | | Q9Y6D6 | ARFGEF1 | |
| *191* | [trinucleotide repeat-containing gene 6C protein isoform X6](http://blast.ncbi.nlm.nih.gov/Blast.cgi#alnHdr_578831201) | | | Q9HCJ0 | TNRC6C | |
| *192* | [chromodomain-helicase-DNA-binding protein 5](http://blast.ncbi.nlm.nih.gov/Blast.cgi#alnHdr_24308089) | | | Q8TDI0 | CHD5 | |
| *193* | [GRB2-related adapter protein 2 isoform 3](http://blast.ncbi.nlm.nih.gov/Blast.cgi#alnHdr_625180318) | | | O75791 | GRAP2 | |
| *194* | [coagulation factor IX isoform X3](http://blast.ncbi.nlm.nih.gov/Blast.cgi#alnHdr_578838643) | | | P00740 | F9 | |
| *195* | [acyl-CoA synthetase short-chain family member 3, mitochondrial isoform X2](http://blast.ncbi.nlm.nih.gov/Blast.cgi#alnHdr_530400836) | | | Q9H6R3 | [ACSS3](http://www.uniprot.org/uniprot/Q9H6R3) | |
| *196* | [tectonin beta-propeller repeat-containing protein 2 isoform 1](http://blast.ncbi.nlm.nih.gov/Blast.cgi#alnHdr_289547515) | | | Q7Z6L1 | [TECPR1](http://www.uniprot.org/uniprot/Q7Z6L1) | |
| *197* | [cytochrome c oxidase assembly factor 6 homolog isoform 1](http://blast.ncbi.nlm.nih.gov/Blast.cgi#alnHdr_61175258) | | | Q5JTJ3 | COA6 | |
| *198* | [lysosomal thioesterase PPT2 isoform a precursor](http://blast.ncbi.nlm.nih.gov/Blast.cgi#alnHdr_323362946) | | | Q9UMR5 | PPT2 | |
| *199* | [protein unc-93 homolog B1](http://blast.ncbi.nlm.nih.gov/Blast.cgi#alnHdr_45580709) | | | Q9H1C4 | UNC93B1 | |
| *200* | [eukaryotic translation initiation factor 4B isoform 2](http://blast.ncbi.nlm.nih.gov/Blast.cgi#alnHdr_50053795) | | | P23588 | [EIF4B](http://www.uniprot.org/uniprot/P23588) | |
| *201* | [coiled-coil domain-containing protein 27](http://blast.ncbi.nlm.nih.gov/Blast.cgi#alnHdr_217035164) | | | Q2M243 | [CCDC27](http://www.uniprot.org/uniprot/Q2M243) | |
| *202* | [rab GTPase-activating protein 1-like isoform A](http://blast.ncbi.nlm.nih.gov/Blast.cgi#alnHdr_78217386) | | | Q5R372 | [RABGAP1L](http://www.uniprot.org/uniprot/B7ZAP0) | |
| *203* | [mis18-binding protein 1](http://blast.ncbi.nlm.nih.gov/Blast.cgi#alnHdr_42415492) | | | Q6P0N0 | [MIS18BP1](http://www.uniprot.org/uniprot/Q6P0N0) | |
| *204* | [myotubularin-related protein 4](http://blast.ncbi.nlm.nih.gov/Blast.cgi#alnHdr_217272865) | | | Q9NYA4 | [MTMR4](http://www.uniprot.org/uniprot/Q9NYA4) | |
| *205* | [neurobeachin-like protein 1 isoform X3](http://blast.ncbi.nlm.nih.gov/Blast.cgi#alnHdr_530370825) | | | Q6ZS30 | [NBEAL1](http://www.uniprot.org/uniprot/Q6ZS30) |  |

| **B.** |  |  |  |  |
| --- | --- | --- | --- | --- |
| *No.* | *Protein* |  | *UniProt accession number* | *Gene ID* |
| *1* | [C2 domain-containing protein 3 isoform X5](http://blast.ncbi.nlm.nih.gov/Blast.cgi#alnHdr_530396574) | Q4AC94 | | [C2CD3](http://www.uniprot.org/uniprot/Q4AC94) |
| *2* | [olfactory receptor 56B1](http://blast.ncbi.nlm.nih.gov/Blast.cgi#alnHdr_52353340) | Q8NGI3 | | OR56B1 |
| *3* | [28S ribosomal protein S11, mitochondrial isoform X2](http://blast.ncbi.nlm.nih.gov/Blast.cgi#alnHdr_530407274) | P82912 | | MRPS11 |
| *4* | [zinc finger protein 891 isoform X1](http://blast.ncbi.nlm.nih.gov/Blast.cgi#alnHdr_578824662) | A8MT65 | | ZNF891 |
| *5* | [lysyl oxidase homolog 4 precursor](http://blast.ncbi.nlm.nih.gov/Blast.cgi#alnHdr_67782349) | Q96JB6 | | LOXL4 |
| *6* | [AT-rich interactive domain-containing protein 2 isoform X2](http://blast.ncbi.nlm.nih.gov/Blast.cgi#alnHdr_578823400) | Q68CP9 | | ARID2 |
| *7* | [rho GTPase-activating protein 27 isoform c](http://blast.ncbi.nlm.nih.gov/Blast.cgi#alnHdr_226817316) | Q6ZUM4 | | ARHGAP27 |
| *8* | [phosphatidylinositol 4-phosphate 3-kinase C2 domain-containing subunit gamma isoform 2](http://blast.ncbi.nlm.nih.gov/Blast.cgi#alnHdr_571026670) | O75747 | | [PIK3C2G](http://www.uniprot.org/uniprot/O75747) |
| *9* | [FAD synthase isoform 4](http://blast.ncbi.nlm.nih.gov/Blast.cgi#alnHdr_296434312) | Q8NFF5 | | [FLAD1](http://www.uniprot.org/uniprot/Q8NFF5) |
| *10* | [HSPB1-associated protein 1](http://blast.ncbi.nlm.nih.gov/Blast.cgi#alnHdr_21314714) | Q96EW2 | | HSPBAP1 |
| *11* | [serine protease inhibitor Kazal-type 14 precursor](http://blast.ncbi.nlm.nih.gov/Blast.cgi#alnHdr_47679099) | Q6IE38 | | SPINK14 |
| *12* | [MLN64 N-terminal domain homolog isoform X2](http://blast.ncbi.nlm.nih.gov/Blast.cgi#alnHdr_530385052) | O95772 | | STARD3NL |
| *13* | [stAR-related lipid transfer protein 3 isoform X4](http://blast.ncbi.nlm.nih.gov/Blast.cgi#alnHdr_578830277) | J3QLS1 | | STARD3 |
| *14* | [glial fibrillary acidic protein isoform 2](http://blast.ncbi.nlm.nih.gov/Blast.cgi#alnHdr_196115290) | P14136 | | GFAP |
| *15* | [POU domain, class 3, transcription factor 3](http://blast.ncbi.nlm.nih.gov/Blast.cgi#alnHdr_5453936) | P20264 | | POU3F3 |
| *16* | [actin-binding LIM protein 1 isoform X13](http://blast.ncbi.nlm.nih.gov/Blast.cgi#alnHdr_530393688) | O14639 | | ABLIM1 |
| *17* | [TPR and ankyrin repeat-containing protein 1](http://blast.ncbi.nlm.nih.gov/Blast.cgi#alnHdr_257467636) | O15050 | | TRANK1 |
| *18* | [AFG3-like protein 2](http://blast.ncbi.nlm.nih.gov/Blast.cgi#alnHdr_300192933) | Q9Y4W6 | | AFG3L2 |
| *19* | [voltage-dependent calcium channel subunit alpha-2/delta-3 isoform X1](http://blast.ncbi.nlm.nih.gov/Blast.cgi#alnHdr_530372845) | Q8IZS8 | | CACNA2D3 |
| *20* | [galectin-10](http://blast.ncbi.nlm.nih.gov/Blast.cgi#alnHdr_20357559) | Q05315 | | CLC |
| *21* | [gonadotropin-releasing hormone receptor isoform 1](http://blast.ncbi.nlm.nih.gov/Blast.cgi#alnHdr_4504059) | P30968 | | GNRHR |
| *22* | [dipeptidyl peptidase 2 isoform X2](http://blast.ncbi.nlm.nih.gov/Blast.cgi#alnHdr_578817401) | Q9UHL4 | | DPP7 |
| *23* | [haloacid dehalogenase-like hydrolase domain-containing protein 3](http://blast.ncbi.nlm.nih.gov/Blast.cgi#alnHdr_13654294) | Q9BSH5 | | HDHD3 |
| *24* | [PR domain zinc finger protein 14](http://blast.ncbi.nlm.nih.gov/Blast.cgi#alnHdr_13375636) | Q9GZV8 | | PRDM14 |
| *25* | [LIM domain and actin-binding protein 1 isoform 3](http://blast.ncbi.nlm.nih.gov/Blast.cgi#alnHdr_165905591) | Q9UHB6 | | LIMA1 |
| *26* | [rab11 family-interacting protein 2](http://blast.ncbi.nlm.nih.gov/Blast.cgi#alnHdr_7662394) | Q7L804 | | RAB11FIP2 |
| *27* | actin | Q5VVB8 | | TMEM244 |
| *28* | [NUAK family SNF1-like kinase 2 isoform X1](http://blast.ncbi.nlm.nih.gov/Blast.cgi#alnHdr_530365437) | Q9H093 | | NUAK2 |
| *29* | [E3 ubiquitin-protein ligase MYCBP2](http://blast.ncbi.nlm.nih.gov/Blast.cgi#alnHdr_291190787) | O75592 | | MYCBP2 |
| *30* | [mediator of RNA polymerase II transcription subunit 13-like isoform X3](http://blast.ncbi.nlm.nih.gov/Blast.cgi#alnHdr_578823481) | Q71F56 | | MED13L |
| *31* | [gamma-glutamyltransferase 5 isoform 4](http://blast.ncbi.nlm.nih.gov/Blast.cgi#alnHdr_699045633) | P36269 | | GGT5 |
| *32* | [semaphorin-4C isoform X1](http://blast.ncbi.nlm.nih.gov/Blast.cgi#alnHdr_578804520) | Q9C0C4 | | SEMA4C |
| *33* | [SPOC domain-containing protein 1 isoform 3](http://blast.ncbi.nlm.nih.gov/Blast.cgi#alnHdr_530354695) | Q6ZMY3 | | SPOCD1 |
| *34* | [IQ domain-containing protein F6](http://blast.ncbi.nlm.nih.gov/Blast.cgi#alnHdr_254028213) | A8MYZ5 | | IQCF6 |
| *35* | [glutamate receptor ionotropic, delta-1 isoform X1](http://blast.ncbi.nlm.nih.gov/Blast.cgi#alnHdr_578819344) | Q9ULK0 | | [GRID1](http://www.uniprot.org/uniprot/Q9ULK0) |
| *36* | [ATP-dependent RNA helicase DDX42](http://blast.ncbi.nlm.nih.gov/Blast.cgi#alnHdr_45446743) | Q86XP3 | | DDX42 |
| *37* | [voltage-dependent P/Q-type calcium channel subunit alpha-1A isoform 3](http://blast.ncbi.nlm.nih.gov/Blast.cgi#alnHdr_187828880) | O00555 | | CACNA1A |
| *38* | [CUE domain-containing protein 2](http://blast.ncbi.nlm.nih.gov/Blast.cgi#alnHdr_148596996) | Q9H467 | | CUEDC2 |
| *39* | [keratin, type II cuticular Hb5 isoform 2](http://blast.ncbi.nlm.nih.gov/Blast.cgi#alnHdr_664806102) | P78386 | | KRT85 |
| *40* | [growth arrest-specific protein 8 isoform b](http://blast.ncbi.nlm.nih.gov/Blast.cgi#alnHdr_555290160) | O95995 | | GAS8 |
| *41* | [keratin, type II cuticular Hb3 isoform X1](http://blast.ncbi.nlm.nih.gov/Blast.cgi#alnHdr_530400232) | P78385 | | KRT83 |
| *42* | [peptidyl-prolyl cis-trans isomerase FKBP4](http://blast.ncbi.nlm.nih.gov/Blast.cgi#alnHdr_4503729) | Q02790 | | FKBP4 |
| *43* | [keratin, type II cuticular Hb6](http://blast.ncbi.nlm.nih.gov/Blast.cgi#alnHdr_14318422) | O43790 | | KRT86 |
| *44* | [keratin, type II cuticular Hb1](http://blast.ncbi.nlm.nih.gov/Blast.cgi#alnHdr_169790853) | Q14533 | | KRT81 |
| *45* | [piggyBac transposable element-derived protein 5](http://blast.ncbi.nlm.nih.gov/Blast.cgi#alnHdr_385198065) | Q8N414 | | PGBD5 |
| *46* | [E3 ubiquitin/ISG15 ligase TRIM25](http://blast.ncbi.nlm.nih.gov/Blast.cgi#alnHdr_68160937) | Q14258 | | TRIM25 |
| *47* | [serine/threonine-protein kinase D1](http://blast.ncbi.nlm.nih.gov/Blast.cgi#alnHdr_115529463) | Q15139 | | PRKD1 |
| *48* | [AP-3 complex subunit delta-1 isoform 2](http://blast.ncbi.nlm.nih.gov/Blast.cgi#alnHdr_117553580) | O14617 | | AP3D1 |
| *49* | [Down syndrome cell adhesion molecule-like protein 1](http://blast.ncbi.nlm.nih.gov/Blast.cgi#alnHdr_21359935) | Q8TD84 | | DSCAML1 |
| *50* | [spectrin alpha chain, non-erythrocytic 1 isoform 3](http://blast.ncbi.nlm.nih.gov/Blast.cgi#alnHdr_306966132) | Q13813 | | SPTAN1 |
| *51* | [serine/threonine-protein kinase SMG1 isoform X4](http://blast.ncbi.nlm.nih.gov/Blast.cgi#alnHdr_530407769) | Q96Q15 | | SMG1 |
| *52* | [brain-enriched guanylate kinase-associated protein isoform X4](http://blast.ncbi.nlm.nih.gov/Blast.cgi#alnHdr_578826012) | Q9BUH8 | | BEGAIN |
| *53* | [solute carrier family 28 member 3](http://blast.ncbi.nlm.nih.gov/Blast.cgi#alnHdr_11545853) | Q9HAS3 | | SLC28A3 |
| *54* | [lysosomal alpha-mannosidase isoform 2 precursor](http://blast.ncbi.nlm.nih.gov/Blast.cgi#alnHdr_291045220) | O00754 | | [MAN2B1](http://www.google.com/url?sa=t&rct=j&q=&esrc=s&source=web&cd=1&ved=0ahUKEwjUyePg_JXLAhXDCJoKHYCxA5AQFgghMAA&url=http%253A%252F%252Fwww.uniprot.org%252Funiprot%252FO00754&usg=AFQjCNH1W7E8r2fup0bEQtj6Y2lnZR3ebg&sig2=MMKqYz8RtHI69n3ZR94UkA&bvm=bv.115339255,d.bGs&cad=rja) |
| *55* | [interferon-inducible GTPase 5](http://blast.ncbi.nlm.nih.gov/Blast.cgi#alnHdr_10257429) | Q6NXR0 | | IRGC |
| *56* | [leukocyte immunoglobulin-like receptor subfamily B member 1 isoform 6 precursor](http://blast.ncbi.nlm.nih.gov/Blast.cgi#alnHdr_612149799) | Q8NHL6 | | LILRB1 |
| *57* | [basal body-orientation factor 1 isoform X6](http://blast.ncbi.nlm.nih.gov/Blast.cgi#alnHdr_530404688) | Q8ND07 | | CCDC176 |
| *58* | [coiled-coil domain-containing protein 170 isoform X1](http://blast.ncbi.nlm.nih.gov/Blast.cgi#alnHdr_578812980) | Q8IYT3 | | CCDC170 |
| *59* | [nesprin-1 isoform 2](http://blast.ncbi.nlm.nih.gov/Blast.cgi#alnHdr_23097308) | Q8NF91 | | SYNE1 |
| *60* | [zinc finger protein 419 isoform 10](http://blast.ncbi.nlm.nih.gov/Blast.cgi#alnHdr_619328927) | Q96HQ0 | | ZNF419 |
| *61* | [radial spoke head protein 9 homolog isoform X2](http://blast.ncbi.nlm.nih.gov/Blast.cgi#alnHdr_578811474) | Q9H1X1 | | RSPH9 |
| *62* | [prostaglandin E2 receptor EP4 subtype isoform X2](http://blast.ncbi.nlm.nih.gov/Blast.cgi#alnHdr_530378866) | P35408 | | PTGER4 |
| *63* | [tripartite motif-containing protein 16-like protein](http://blast.ncbi.nlm.nih.gov/Blast.cgi#alnHdr_82617564) | Q309B1 | | TRIM16L |
| *64* | [carbohydrate sulfotransferase 12](http://blast.ncbi.nlm.nih.gov/Blast.cgi#alnHdr_8922112) | Q9NRB3 | | CHST12 |
| *65* | [leukocyte immunoglobulin-like receptor subfamily B member 4 isoform X5](http://blast.ncbi.nlm.nih.gov/Blast.cgi#alnHdr_530431848) | Q8NHJ6 | | LILRB4 |
| *66* | [beta-galactoside alpha-2,6-sialyltransferase 2 isoform b](http://blast.ncbi.nlm.nih.gov/Blast.cgi#alnHdr_215272347) | Q96JF0 | | ST6GAL2 |
| *67* | [HAUS augmin-like complex subunit 5 isoform X2](http://blast.ncbi.nlm.nih.gov/Blast.cgi#alnHdr_578834213) | O94927 | | HAUS5 |
| *68* | [tripartite motif-containing protein 16](http://blast.ncbi.nlm.nih.gov/Blast.cgi#alnHdr_48255913) | O95361 | | TRIM16 |
| *69* | [tetratricopeptide repeat protein 24](http://blast.ncbi.nlm.nih.gov/Blast.cgi#alnHdr_282165719) | A2A3L6 | | TTC24 |
| *70* | [anthrax toxin receptor-like isoform X1](http://blast.ncbi.nlm.nih.gov/Blast.cgi#alnHdr_578819056) | A6NF34 | | ANTXRL |
| *71* | [peptidyl-prolyl cis-trans isomerase G](http://blast.ncbi.nlm.nih.gov/Blast.cgi#alnHdr_42560244) | Q13427 | | PPIG |
| *72* | [cadherin-like and PC-esterase domain-containing protein 1 isoform 2 precursor](http://blast.ncbi.nlm.nih.gov/Blast.cgi#alnHdr_157671947) | A4D0V7 | | CPED1 |
| *73* | [valine--tRNA ligase, mitochondrial isoform 3](http://blast.ncbi.nlm.nih.gov/Blast.cgi#alnHdr_268370295) | Q5ST30 | | VARS2 |
| *74* | [unconventional myosin-Ic isoform c](http://blast.ncbi.nlm.nih.gov/Blast.cgi#alnHdr_124494240) | O00159 | | MYO1C |
| *75* | [lysine-specific demethylase hairless isoform b](http://blast.ncbi.nlm.nih.gov/Blast.cgi#alnHdr_22547207) | O43593 | | HR |
| *76* | [astrotactin-2 isoform a precursor](http://blast.ncbi.nlm.nih.gov/Blast.cgi#alnHdr_46488915) | O75129 | | ASTN2 |
| *77* | [dedicator of cytokinesis protein 7 isoform X3](http://blast.ncbi.nlm.nih.gov/Blast.cgi#alnHdr_578799994) | Q96N67 | | DOCK7 |
| *78* | [microtubule cross-linking factor 1](http://blast.ncbi.nlm.nih.gov/Blast.cgi#alnHdr_163644316) | Q9Y4B5 | | MTCL1 |
| *79* | [voltage-dependent L-type calcium channel subunit alpha-1S](http://blast.ncbi.nlm.nih.gov/Blast.cgi#alnHdr_110349767) | Q13698 | | CACNA1S |
| *80* | [dedicator of cytokinesis protein 8 isoform 2](http://blast.ncbi.nlm.nih.gov/Blast.cgi#alnHdr_299473744) | Q8NF50 | | DOCK8 |
| *81* | [dynein heavy chain 9, axonemal isoform X3](http://blast.ncbi.nlm.nih.gov/Blast.cgi#alnHdr_578829754) | Q9NYC9 | | DNAH9 |
| *82* | [sal-like protein 2 isoform c](http://blast.ncbi.nlm.nih.gov/Blast.cgi#alnHdr_612339330) | Q9Y467 | | SALL2 |
| *83* | [zinc finger protein 451 isoform X2](http://blast.ncbi.nlm.nih.gov/Blast.cgi#alnHdr_530381758) | Q9Y4E5 | | ZNF451 |
| *84* | [periplakin](http://blast.ncbi.nlm.nih.gov/Blast.cgi#alnHdr_45439327) | O60437 | | PPL |
| *85* | [tyrosine-protein phosphatase non-receptor type 13 isoform 3](http://blast.ncbi.nlm.nih.gov/Blast.cgi#alnHdr_18375648) | Q12923 | | PTPN13 |
| *86* | [uncharacterized protein C11orf85 isoform X8](http://blast.ncbi.nlm.nih.gov/Blast.cgi#alnHdr_578821307) | Q3KP22 | | C11ORF85 |
| *87* | [D-amino-acid oxidase isoform X3](http://blast.ncbi.nlm.nih.gov/Blast.cgi#alnHdr_578823391) | P14920 | | DAO |
| *88* | [high affinity immunoglobulin epsilon receptor subunit alpha precursor](http://blast.ncbi.nlm.nih.gov/Blast.cgi#alnHdr_4503675) | P12319 | | FCER1A |
| *89* | [chymotrypsin-like elastase family member 2A preproprotein](http://blast.ncbi.nlm.nih.gov/Blast.cgi#alnHdr_15559207) | P08217 | | CELA2A |
| *90* | [inorganic pyrophosphatase](http://blast.ncbi.nlm.nih.gov/Blast.cgi#alnHdr_11056044) | Q15181 | | PPA1 |
| *91* | [fas apoptotic inhibitory molecule 3 isoform X2](http://blast.ncbi.nlm.nih.gov/Blast.cgi#alnHdr_530366662) | O60667 | | FCMR |
| *92* | [interferon gamma receptor 2 precursor](http://blast.ncbi.nlm.nih.gov/Blast.cgi#alnHdr_47419934) | P38484 | | IFNGR2 |
| *93* | [inhibin alpha chain preproprotein](http://blast.ncbi.nlm.nih.gov/Blast.cgi#alnHdr_4504697) | P05111 | | INHA |
| *94* | [pygopus homolog 2](http://blast.ncbi.nlm.nih.gov/Blast.cgi#alnHdr_23510333) | Q9BRQ0 | | PYGO2 |
| *95* | [mitochondrial carrier homolog 1 isoform X2](http://blast.ncbi.nlm.nih.gov/Blast.cgi#alnHdr_530381726) | Q9NZJ7 | | MTCH1 |
| *96* | [pygopus homolog 1](http://blast.ncbi.nlm.nih.gov/Blast.cgi#alnHdr_30911103) | Q9Y3Y4 | | PYGO1 |
| *97* | [coiled-coil domain-containing protein 47 precursor](http://blast.ncbi.nlm.nih.gov/Blast.cgi#alnHdr_171906582) | Q96A33 | | CCDC47 |
| *98* | [POTE ankyrin domain family member C](http://blast.ncbi.nlm.nih.gov/Blast.cgi#alnHdr_212549546) | B2RU33 | | POTEC |
| *99* | [caspase recruitment domain-containing protein 6](http://blast.ncbi.nlm.nih.gov/Blast.cgi#alnHdr_16554564) | Q9BX69 | | CARD6 |
| *100* | [1-phosphatidylinositol 4,5-bisphosphate phosphodiesterase gamma-2](http://blast.ncbi.nlm.nih.gov/Blast.cgi#alnHdr_117320537) | P16885 | | PLCG2 |
| *101* | [1-phosphatidylinositol 4,5-bisphosphate phosphodiesterase gamma-1 isoform b](http://blast.ncbi.nlm.nih.gov/Blast.cgi#alnHdr_33598946) | P19174 | | PLCG1 |
| *102* | [heat shock factor protein 5](http://blast.ncbi.nlm.nih.gov/Blast.cgi#alnHdr_612407780) | Q4G112 | | HSF5 |
| *103* | [prolyl 4-hydroxylase subunit alpha-2 isoform 2 precursor](http://blast.ncbi.nlm.nih.gov/Blast.cgi#alnHdr_63252891) | O15460 | | P4HA2 |
| *104* | [sprT-like domain-containing protein Spartan isoform c](http://blast.ncbi.nlm.nih.gov/Blast.cgi#alnHdr_387762597) | Q9H040 | | SPRTN |
| *105* | [fibroblast growth factor receptor substrate 3 isoform X1](http://blast.ncbi.nlm.nih.gov/Blast.cgi#alnHdr_578811367) | O43559 | | FRS3 |
| *106* | [hyccin isoform X3](http://blast.ncbi.nlm.nih.gov/Blast.cgi#alnHdr_578813689) | Q9BYI3 | | FAM126A |
| *107* | [cerebral cavernous malformations 2 protein-like](http://blast.ncbi.nlm.nih.gov/Blast.cgi#alnHdr_31542255) | Q9NUG4 | | CCM2L |
| *108* | [cytoplasmic polyadenylation element-binding protein 2 isoform A](http://blast.ncbi.nlm.nih.gov/Blast.cgi#alnHdr_293651595) | Q7Z5Q1 | | CPEB2 |
| *109* | [zinc finger and BTB domain-containing protein 16](http://blast.ncbi.nlm.nih.gov/Blast.cgi#alnHdr_21359888) | Q05516 | | ZBTB16 |
| *110* | [protein kintoun isoform 2](http://blast.ncbi.nlm.nih.gov/Blast.cgi#alnHdr_145580586) | Q9NVR5 | | DNAAF2 |
| *111* | [thyroid receptor-interacting protein 11 isoform X3](http://blast.ncbi.nlm.nih.gov/Blast.cgi#alnHdr_530404933) | Q15643 | | TRIP11 |
| *112* | [DNA mismatch repair protein Msh6 isoform 3](http://blast.ncbi.nlm.nih.gov/Blast.cgi#alnHdr_528078300) | P52701 | | MSH6 |
| *113* | [3'(2'),5'-bisphosphate nucleotidase 1 isoform X7](http://blast.ncbi.nlm.nih.gov/Blast.cgi#alnHdr_530365939) | O95861 | | BPNT1 |
| *114* | [Hermansky-Pudlak syndrome 5 protein isoform b](http://blast.ncbi.nlm.nih.gov/Blast.cgi#alnHdr_31657125) | Q9UPZ3 | | HPS5 |
| *115* | [metabotropic glutamate receptor 5 isoform a precursor](http://blast.ncbi.nlm.nih.gov/Blast.cgi#alnHdr_219842343) | P41594 | | GRM5 |
| *116* | [rho guanine nucleotide exchange factor 10-like protein isoform X6](http://blast.ncbi.nlm.nih.gov/Blast.cgi#alnHdr_578799368) | Q9HCE6 | | ARHGEF10 |
| *117* | [neurogenic locus notch homolog protein 2 isoform 2 precursor](http://blast.ncbi.nlm.nih.gov/Blast.cgi#alnHdr_317008611) | Q04721 | | NOTCH2 |
| *118* | [glutamate receptor ionotropic, NMDA 2B isoform X1](http://blast.ncbi.nlm.nih.gov/Blast.cgi#alnHdr_530399104) | Q13224 | | GRIN2B |
| *119* | [protein SMG9](http://blast.ncbi.nlm.nih.gov/Blast.cgi#alnHdr_145301567) | Q9H0W8 | | SMG9 |
| *120* | [hyaluronan synthase 2](http://blast.ncbi.nlm.nih.gov/Blast.cgi#alnHdr_4885391) | Q92819 | | HAS2 |
| *121* | [zinc finger protein 837](http://blast.ncbi.nlm.nih.gov/Blast.cgi#alnHdr_193082980) | Q96EG3 | | ZNF837 |
| *122* | [zinc finger protein 786](http://blast.ncbi.nlm.nih.gov/Blast.cgi#alnHdr_170932473) | Q8N393 | | ZNF786 |
| *123* | [glutamate receptor ionotropic, NMDA 2D precursor](http://blast.ncbi.nlm.nih.gov/Blast.cgi#alnHdr_153946391) | O15399 | | GRIN2D |
| *124* | [DNA (cytosine-5)-methyltransferase 3-like](http://blast.ncbi.nlm.nih.gov/Blast.cgi#alnHdr_578836316) | Q9UJW3 | | DNMT3L |
| *125* | [tumor necrosis factor ligand superfamily member 14 isoform 2](http://blast.ncbi.nlm.nih.gov/Blast.cgi#alnHdr_291045244) | O43557 | | TNFSF14 |
| *126* | [solute carrier family 25 member 44 isoform 2](http://blast.ncbi.nlm.nih.gov/Blast.cgi#alnHdr_45237193) | Q96H78 | | SLC25A44 |
| *127* | [dual specificity protein phosphatase 5](http://blast.ncbi.nlm.nih.gov/Blast.cgi#alnHdr_62865890) | Q16690 | | DUSP5 |
| *128* | [zinc finger protein 302 isoform c](http://blast.ncbi.nlm.nih.gov/Blast.cgi#alnHdr_59850649) | Q9NR11 | | ZNF302 |
| *129* | [stromelysin-3 preproprotein](http://blast.ncbi.nlm.nih.gov/Blast.cgi#alnHdr_58331148) | P24347 | | MMP11 |
| *130* | [vitamin K-dependent gamma-carboxylase isoform 2](http://blast.ncbi.nlm.nih.gov/Blast.cgi#alnHdr_214010149) | P38435 | | GGCX |
| *131* | [stonin-2 isoform X3](http://blast.ncbi.nlm.nih.gov/Blast.cgi#alnHdr_578826198) | Q8WXE9 | | STON2 |
| *132* | [BRCA1-associated ATM activator 1 isoform X3](http://blast.ncbi.nlm.nih.gov/Blast.cgi#alnHdr_530384539) | Q6PJG6 | | BRAT1 |
| *133* | [collagen alpha-5(VI) chain isoform 2 precursor](http://blast.ncbi.nlm.nih.gov/Blast.cgi#alnHdr_183583553) | A8TX70 | | COL6A5 |
| *134* | [bone marrow proteoglycan isoform 2 preproprotein](http://blast.ncbi.nlm.nih.gov/Blast.cgi#alnHdr_342837699) | P13727 | | PRG2 |
| *135* | [solute carrier family 35 member E3 isoform X1](http://blast.ncbi.nlm.nih.gov/Blast.cgi#alnHdr_530400532) | Q7Z769 | | SLC35E3 |
| *136* | [beta-1,4-galactosyltransferase 6 isoform X3](http://blast.ncbi.nlm.nih.gov/Blast.cgi#alnHdr_578832783) | Q9UBX8 | | B4GALT6 |
| *137* | [autophagy-related protein 16-2 isoform X5](http://blast.ncbi.nlm.nih.gov/Blast.cgi#alnHdr_578821841) | Q8NAA4 | | ATG16L2 |
| *138* | [2-oxoisovalerate dehydrogenase subunit beta, mitochondrial isoform X1](http://blast.ncbi.nlm.nih.gov/Blast.cgi#alnHdr_530383218) | P21953 | | BCKDHB |
| *139* | [zinc finger and BTB domain-containing protein 38](http://blast.ncbi.nlm.nih.gov/Blast.cgi#alnHdr_148276990) | Q8NAP3 | | ZBTB38 |
| *140* | [phosphatidylinositol-glycan biosynthesis class X protein isoform 2 precursor](http://blast.ncbi.nlm.nih.gov/Blast.cgi#alnHdr_261490706) | Q8TBF5 | | PIGX |
| *141* | [calcium/calmodulin-dependent protein kinase type II subunit alpha isoform 1](http://blast.ncbi.nlm.nih.gov/Blast.cgi#alnHdr_25952114) | Q9UQM7 | | CAMK2A |
| *142* | [methyltransferase-like protein 7A precursor](http://blast.ncbi.nlm.nih.gov/Blast.cgi#alnHdr_89145417) | Q9H8H3 | | METTL7A |
| *143* | [STAM-binding protein isoform X4](http://blast.ncbi.nlm.nih.gov/Blast.cgi#alnHdr_530367130) | O95630 | | STAMBP |
| *144* | [24-hydroxycholesterol 7-alpha-hydroxylase isoform 3](http://blast.ncbi.nlm.nih.gov/Blast.cgi#alnHdr_525313660) | Q9NYL5 | | CYP39A1 |
| *145* | [AMSH-like protease](http://blast.ncbi.nlm.nih.gov/Blast.cgi#alnHdr_33147080) | Q96FJ0 | | STAMBPL1 |
| *146* | [transmembrane 9 superfamily member 3 precursor](http://blast.ncbi.nlm.nih.gov/Blast.cgi#alnHdr_190194386) | Q9HD45 | | TM9SF3 |
| *147* | [disintegrin and metalloproteinase domain-containing protein 32 isoform X2](http://blast.ncbi.nlm.nih.gov/Blast.cgi#alnHdr_578839796) | Q8TC27 | | ADAM32 |
| *148* | [nuclear autoantigen Sp-100 isoform 3](http://blast.ncbi.nlm.nih.gov/Blast.cgi#alnHdr_331999992) | P23497 | | SP100 |
| *149* | [zinc fingers and homeoboxes protein 2](http://blast.ncbi.nlm.nih.gov/Blast.cgi#alnHdr_7662342) | Q9Y6X8 | | ZHX2 |
| *150* | [mdm2-binding protein](http://blast.ncbi.nlm.nih.gov/Blast.cgi#alnHdr_21630257) | Q96DY7 | | MTBP |
| *151* | [zinc finger E-box-binding homeobox 1 isoform e](http://blast.ncbi.nlm.nih.gov/Blast.cgi#alnHdr_291575190) | P37275 | | ZEB1 |
| *152* | [protogenin isoform X3](http://blast.ncbi.nlm.nih.gov/Blast.cgi#alnHdr_578826833) | Q2VWP7 | | PRTG |
| *153* | [regulator of nonsense transcripts 1 isoform 1](http://blast.ncbi.nlm.nih.gov/Blast.cgi#alnHdr_662033888) | Q92900 | | UPF1 |
| *154* | [mitochondrial fission regulator 2](http://blast.ncbi.nlm.nih.gov/Blast.cgi#alnHdr_39653315) | Q6P444 | | MTFR2 |
| *155* | [serine incorporator 1 precursor](http://blast.ncbi.nlm.nih.gov/Blast.cgi#alnHdr_24308213) | Q9NRX5 | | SERINC1 |
| *156* | [myotubularin-related protein 6 isoform X1](http://blast.ncbi.nlm.nih.gov/Blast.cgi#alnHdr_578825077) | Q9Y217 | | MTMR6 |
| *157* | [retinoic acid receptor gamma isoform X1](http://blast.ncbi.nlm.nih.gov/Blast.cgi#alnHdr_530400634) | P13631 | | RARG |
| *158* | [tyrosine-protein kinase Tec isoform X1](http://blast.ncbi.nlm.nih.gov/Blast.cgi#alnHdr_530376704) | P42680 | | TEC |
| *159* | [ankyrin repeat domain-containing protein 6 isoform X6](http://blast.ncbi.nlm.nih.gov/Blast.cgi#alnHdr_530383069) | Q9Y2G4 | | ANKRD6 |
| *160* | [rho GTPase-activating protein 9 isoform 2](http://blast.ncbi.nlm.nih.gov/Blast.cgi#alnHdr_122939151) | Q9BRR9 | | ARHGAP9 |
| *161* | [scavenger receptor cysteine-rich type 1 protein M130 isoform b precursor](http://blast.ncbi.nlm.nih.gov/Blast.cgi#alnHdr_344179112) | Q86VB7 | | CD163 |
| *162* | [brefeldin A-inhibited guanine nucleotide-exchange protein 2 isoform X2](http://blast.ncbi.nlm.nih.gov/Blast.cgi#alnHdr_578835710) | Q9Y6D5 | | ARFGEF2 |
| *163* | [tectonin beta-propeller repeat-containing protein 2 isoform 2](http://blast.ncbi.nlm.nih.gov/Blast.cgi#alnHdr_289547517) | O15040 | | TECPR2 |
| *164* | [spermatogenesis-associated protein 31E1](http://blast.ncbi.nlm.nih.gov/Blast.cgi#alnHdr_155029550) | Q6ZUB1 | | SPATA31E1 |
| *165* | [E3 ubiquitin-protein ligase SHPRH isoform X8](http://blast.ncbi.nlm.nih.gov/Blast.cgi#alnHdr_578812645) | Q149N8 | | SHPRH |
| *166* | [brefeldin A-inhibited guanine nucleotide-exchange protein 1 isoform X4](http://blast.ncbi.nlm.nih.gov/Blast.cgi#alnHdr_578815582) | Q9Y6D6 | | ARFGEF1 |
| *167* | [trinucleotide repeat-containing gene 6C protein isoform X6](http://blast.ncbi.nlm.nih.gov/Blast.cgi#alnHdr_578831201) | Q9HCJ0 | | TNRC6C |
| *168* | [chromodomain-helicase-DNA-binding protein 5](http://blast.ncbi.nlm.nih.gov/Blast.cgi#alnHdr_24308089) | Q8TDI0 | | CHD5 |
| *169* | [GRB2-related adapter protein 2 isoform 3](http://blast.ncbi.nlm.nih.gov/Blast.cgi#alnHdr_625180318) | O75791 | | GRAP2 |
| *170* | [coagulation factor IX isoform X3](http://blast.ncbi.nlm.nih.gov/Blast.cgi#alnHdr_578838643) | P00740 | | F9 |
| *171* | [acyl-CoA synthetase short-chain family member 3, mitochondrial isoform X2](http://blast.ncbi.nlm.nih.gov/Blast.cgi#alnHdr_530400836) | Q9H6R3 | | [ACSS3](http://www.uniprot.org/uniprot/Q14DH7) |
| *172* | [tectonin beta-propeller repeat-containing protein 2 isoform 1](http://blast.ncbi.nlm.nih.gov/Blast.cgi#alnHdr_289547515) | Q7Z6L1 | | [TECPR1](http://www.uniprot.org/uniprot/Q7Z6L1) |
| *173* | [cytochrome c oxidase assembly factor 6 homolog isoform 1](http://blast.ncbi.nlm.nih.gov/Blast.cgi#alnHdr_61175258) | Q5JTJ3 | | COA6 |
| *174* | [lysosomal thioesterase PPT2 isoform a precursor](http://blast.ncbi.nlm.nih.gov/Blast.cgi#alnHdr_323362946) | Q9UMR5 | | PPT2 |
| *175* | [protein unc-93 homolog B1](http://blast.ncbi.nlm.nih.gov/Blast.cgi#alnHdr_45580709) | Q9H1C4 | | UNC93B1 |
| *176* | [eukaryotic translation initiation factor 4B isoform 2](http://blast.ncbi.nlm.nih.gov/Blast.cgi#alnHdr_50053795) | P23588 | | [EIF4B](http://www.uniprot.org/uniprot/P23588) |
| *177* | [coiled-coil domain-containing protein 27](http://blast.ncbi.nlm.nih.gov/Blast.cgi#alnHdr_217035164) | Q2M243 | | [CCDC27](http://www.uniprot.org/uniprot/Q2M243) |
| *178* | [rab GTPase-activating protein 1-like isoform A](http://blast.ncbi.nlm.nih.gov/Blast.cgi#alnHdr_78217386) | Q5R372 | | [RABGAP1L](http://www.uniprot.org/uniprot/B7ZAP0) |
| *179* | [mis18-binding protein 1](http://blast.ncbi.nlm.nih.gov/Blast.cgi#alnHdr_42415492) | Q6P0N0 | | [MIS18BP1](http://www.uniprot.org/uniprot/Q6P0N0) |
| *180* | [myotubularin-related protein 4](http://blast.ncbi.nlm.nih.gov/Blast.cgi#alnHdr_217272865) | Q9NYA4 | | [MTMR4](http://www.uniprot.org/uniprot/Q9NYA4) |
| *181* | [neurobeachin-like protein 1 isoform X3](http://blast.ncbi.nlm.nih.gov/Blast.cgi#alnHdr_530370825) | Q6ZS30 | | [NBEAL1](http://www.uniprot.org/uniprot/Q6ZS30) |

| **C*.*** |  |  |  |
| --- | --- | --- | --- |
| *No.* | *Protein* | *UniProt accession numbers* | *Gene ID* |
| *1* | [C2 domain-containing protein 3 isoform X5](http://blast.ncbi.nlm.nih.gov/Blast.cgi#alnHdr_530396574) | Q4AC94 | [C2CD3](http://www.uniprot.org/uniprot/Q4AC94) |
| *2* | [rho GTPase-activating protein 27 isoform c](http://blast.ncbi.nlm.nih.gov/Blast.cgi#alnHdr_226817316) | Q6ZUM4 | ARHGAP27 |
| *3* | [phosphatidylinositol 4-phosphate 3-kinase C2 domain-containing subunit gamma isoform 2](http://blast.ncbi.nlm.nih.gov/Blast.cgi#alnHdr_571026670) | O75747 | PIK3C2G |
| *4* | [FAD synthase isoform 4](http://blast.ncbi.nlm.nih.gov/Blast.cgi#alnHdr_296434312) | Q8NFF5 | [FLAD1](http://www.uniprot.org/uniprot/Q8NFF5) |
| *5* | [HSPB1-associated protein 1](http://blast.ncbi.nlm.nih.gov/Blast.cgi#alnHdr_21314714) | Q96EW2 | HSPBAP1 |
| *6* | [AFG3-like protein 2](http://blast.ncbi.nlm.nih.gov/Blast.cgi#alnHdr_300192933) | Q9Y4W6 | AFG3L2 |
| *7* | [voltage-dependent calcium channel subunit alpha-2/delta-3 isoform X1](http://blast.ncbi.nlm.nih.gov/Blast.cgi#alnHdr_530372845) | Q8IZS8 | CACNA2D3 |
| *8* | [gonadotropin-releasing hormone receptor isoform 1](http://blast.ncbi.nlm.nih.gov/Blast.cgi#alnHdr_4504059) | P30968 | GNRHR |
| *9* | [transmembrane protein 244](http://blast.ncbi.nlm.nih.gov/Blast.cgi#alnHdr_58197570) | Q5VVB8 | TMEM244 |
| *10* | [NUAK family SNF1-like kinase 2 isoform X1](http://blast.ncbi.nlm.nih.gov/Blast.cgi#alnHdr_530365437) | Q9H093 | NUAK2 |
| *11* | [E3 ubiquitin-protein ligase MYCBP2](http://blast.ncbi.nlm.nih.gov/Blast.cgi#alnHdr_291190787) | O75592 | MYCBP2 |
| *12* | [mediator of RNA polymerase II transcription subunit 13-like isoform X3](http://blast.ncbi.nlm.nih.gov/Blast.cgi#alnHdr_578823481) | Q71F56 | MED13L |
| *13* | [IQ domain-containing protein F6](http://blast.ncbi.nlm.nih.gov/Blast.cgi#alnHdr_254028213) | A8MYZ5 | IQCF6 |
| *14* | [glutamate receptor ionotropic, delta-1 isoform X1](http://blast.ncbi.nlm.nih.gov/Blast.cgi#alnHdr_578819344) | Q9ULK0 | [GRID1](http://www.uniprot.org/uniprot/Q9ULK0) |
| *15* | [solute carrier family 28 member 3](http://blast.ncbi.nlm.nih.gov/Blast.cgi#alnHdr_11545853) | Q9HAS3 | SLC28A3 |
| *16* | [lysosomal alpha-mannosidase isoform 2 precursor](http://blast.ncbi.nlm.nih.gov/Blast.cgi#alnHdr_291045220) | O00754 | [MAN2B1](http://www.google.com/url?sa=t&rct=j&q=&esrc=s&source=web&cd=1&ved=0ahUKEwjUyePg_JXLAhXDCJoKHYCxA5AQFgghMAA&url=http%253A%252F%252Fwww.uniprot.org%252Funiprot%252FO00754&usg=AFQjCNH1W7E8r2fup0bEQtj6Y2lnZR3ebg&sig2=MMKqYz8RtHI69n3ZR94UkA&bvm=bv.115339255,d.bGs&cad=rja) |
| *17* | [heat shock factor protein 5](http://blast.ncbi.nlm.nih.gov/Blast.cgi#alnHdr_612407780) | Q4G112 | HSF5 |
| *18* | [prolyl 4-hydroxylase subunit alpha-2 isoform 2 precursor](http://blast.ncbi.nlm.nih.gov/Blast.cgi#alnHdr_63252891) | O15460 | P4HA2 |
| *19* | [sprT-like domain-containing protein Spartan isoform c](http://blast.ncbi.nlm.nih.gov/Blast.cgi#alnHdr_387762597) | Q9H040 | SPRTN |
| *20* | [mitochondrial fission regulator 2](http://blast.ncbi.nlm.nih.gov/Blast.cgi#alnHdr_39653315) | Q6P444 | MTFR2 |
| *21* | [serine incorporator 1 precursor](http://blast.ncbi.nlm.nih.gov/Blast.cgi#alnHdr_24308213) | Q9NRX5 | SERINC1 |
| *22* | [myotubularin-related protein 6 isoform X1](http://blast.ncbi.nlm.nih.gov/Blast.cgi#alnHdr_578825077) | Q9Y217 | MTMR6 |
| *23* | [retinoic acid receptor gamma isoform X1](http://blast.ncbi.nlm.nih.gov/Blast.cgi#alnHdr_530400634) | P13631 | RARG |
| *24* | [tyrosine-protein kinase Tec isoform X1](http://blast.ncbi.nlm.nih.gov/Blast.cgi#alnHdr_530376704) | P42680 | TEC |
| *25* | [ankyrin repeat domain-containing protein 6 isoform X6](http://blast.ncbi.nlm.nih.gov/Blast.cgi#alnHdr_530383069) | Q9Y2G4 | ANKRD6 |
| *26* | [rho GTPase-activating protein 9 isoform 2](http://blast.ncbi.nlm.nih.gov/Blast.cgi#alnHdr_122939151) | Q9BRR9 | ARHGAP9 |
| *27* | [scavenger receptor cysteine-rich type 1 protein M130 isoform b precursor](http://blast.ncbi.nlm.nih.gov/Blast.cgi#alnHdr_344179112) | Q86VB7 | CD163 |
| *28* | [brefeldin A-inhibited guanine nucleotide-exchange protein 2 isoform X2](http://blast.ncbi.nlm.nih.gov/Blast.cgi#alnHdr_578835710) | Q9Y6D5 | ARFGEF2 |
| *29* | [tectonin beta-propeller repeat-containing protein 2 isoform 2](http://blast.ncbi.nlm.nih.gov/Blast.cgi#alnHdr_289547517) | O15040 | TECPR2 |
| *30* | [spermatogenesis-associated protein 31E1](http://blast.ncbi.nlm.nih.gov/Blast.cgi#alnHdr_155029550) | Q6ZUB1 | SPATA31E1 |
| *31* | [E3 ubiquitin-protein ligase SHPRH isoform X8](http://blast.ncbi.nlm.nih.gov/Blast.cgi#alnHdr_578812645) | Q149N8 | SHPRH |
| *32* | [brefeldin A-inhibited guanine nucleotide-exchange protein 1 isoform X4](http://blast.ncbi.nlm.nih.gov/Blast.cgi#alnHdr_578815582) | Q9Y6D6 | ARFGEF1 |
| *33* | [trinucleotide repeat-containing gene 6C protein isoform X6](http://blast.ncbi.nlm.nih.gov/Blast.cgi#alnHdr_578831201) | Q9HCJ0 | TNRC6C |
| *34* | [chromodomain-helicase-DNA-binding protein 5](http://blast.ncbi.nlm.nih.gov/Blast.cgi#alnHdr_24308089) | Q8TDI0 | CHD5 |
| *35* | [GRB2-related adapter protein 2 isoform 3](http://blast.ncbi.nlm.nih.gov/Blast.cgi#alnHdr_625180318) | O75791 | GRAP2 |
| *36* | [coagulation factor IX isoform X3](http://blast.ncbi.nlm.nih.gov/Blast.cgi#alnHdr_578838643) | P00740 | F9 |
| *37* | [acyl-CoA synthetase short-chain family member 3, mitochondrial isoform X2](http://blast.ncbi.nlm.nih.gov/Blast.cgi#alnHdr_530400836) | Q9H6R3 | [ACSS3](http://www.uniprot.org/uniprot/Q14DH7) |
| *38* | [tectonin beta-propeller repeat-containing protein 2 isoform 1](http://blast.ncbi.nlm.nih.gov/Blast.cgi#alnHdr_289547515) | Q7Z6L1 | [TECPR1](http://www.uniprot.org/uniprot/Q7Z6L1) |
| *39* | [cytochrome c oxidase assembly factor 6 homolog isoform 1](http://blast.ncbi.nlm.nih.gov/Blast.cgi#alnHdr_61175258) | Q5JTJ3 | COA6 |
| *40* | [lysosomal thioesterase PPT2 isoform a precursor](http://blast.ncbi.nlm.nih.gov/Blast.cgi#alnHdr_323362946) | Q9UMR5 | PPT2 |
| *41* | [protein unc-93 homolog B1](http://blast.ncbi.nlm.nih.gov/Blast.cgi#alnHdr_45580709) | Q9H1C4 | UNC93B1 |
| *42* | [eukaryotic translation initiation factor 4B isoform 2](http://blast.ncbi.nlm.nih.gov/Blast.cgi#alnHdr_50053795) | P23588 | [EIF4B](http://www.uniprot.org/uniprot/P23588) |
| *43* | [coiled-coil domain-containing protein 27](http://blast.ncbi.nlm.nih.gov/Blast.cgi#alnHdr_217035164) | Q2M243 | [CCDC27](http://www.uniprot.org/uniprot/Q2M243) |
| *44* | [rab GTPase-activating protein 1-like isoform A](http://blast.ncbi.nlm.nih.gov/Blast.cgi#alnHdr_78217386) | Q5R372 | [RABGAP1L](http://www.uniprot.org/uniprot/B7ZAP0) |
| *45* | [mis18-binding protein 1](http://blast.ncbi.nlm.nih.gov/Blast.cgi#alnHdr_42415492) | Q6P0N0 | [MIS18BP1](http://www.uniprot.org/uniprot/Q6P0N0) |
| *46* | [myotubularin-related protein 4](http://blast.ncbi.nlm.nih.gov/Blast.cgi#alnHdr_217272865) | Q9NYA4 | [MTMR4](http://www.uniprot.org/uniprot/Q9NYA4) |
| *47* | [neurobeachin-like protein 1 isoform X3](http://blast.ncbi.nlm.nih.gov/Blast.cgi#alnHdr_530370825) | Q6ZS30 | [NBEAL1](http://www.uniprot.org/uniprot/Q6ZS30) |
