# Supplementary material for: A systems medicine approach for finding target proteins affecting treatment outcomes in patients with non-Hodgkin lymphoma
Source: PLoS One. 2017 Sep 11;12(9):e0183969. doi: 10.1371/journal.pone.0183969 (PMC5593188; doi:10.1371/journal.pone.0183969)
Supplement: S5 Table — (A) Transcriptional regulatory network in embryonic stem cells (Ratio: 4/40 and P-value: 5.42E-04), (B) RhoA signaling (Ratio: 6/122 (0.049) and P-value: 1.08E-03), (C) Adipogenesis pathway (Ratio: 4/134 (0.037) and P-value: 8.97E-03), (D) Telomerase signaling (Ratio: 4/99 (0.04) and P-value: 1.45E-02), and (E) DNA methylation and transcriptional repression signaling (Ratio: 2/20 (0.1) and P-value: 1.51E-02). N: Nucleus, TR: Transcription regulator, C: Cytoplasm, E: Enzyme, PM: Plasma membrane, GPCR: G-protein coupled receptor, P: Phosphatase, and PP: Peptidase. (DOCX) [file pone.0183969.s008.docx]

**S5 Table. Top canonical networks at PR gene signature identified through IPA.** (A) Transcriptional regulatory network in embryonic stem cells (Ratio: 4/40 and *P*-value: 5.42E-04), (B) RhoA signaling (Ratio: 6/122 (0.049) and *P*-value: 1.08E-03), (C) Adipogenesis pathway (Ratio: 4/134 (0.037) and *P*-value: 8.97E-03), (D) Telomerase signaling (Ratio: 4/99 (0.04) and *P*-value: 1.45E-02), and (E) DNA methylation and transcriptional repression signaling (Ratio: 2/20 (0.1) and *P*-value: 1.51E-02). N: Nucleus, TR: Transcription regulator, C: Cytoplasm, E: Enzyme, PM: Plasma membrane, GPCR: G-protein coupled receptor, P: Phosphatase, and PP: Peptidase.

| **A.** |  |  |  |
| --- | --- | --- | --- |
| **Symbol** | **Entrez Gene Name** | **Location** | **Types** |
| HAND1 | Heart and neural crest | N | TR |
| MEIS1 | Meis homeobox 1 | N | TR |
| ONECUT1 | One cut homeobox 1 | N | TR |
| OTX1 | Glutamate receptor | N | TR |

| **B.** |  |  |  |  |
| --- | --- | --- | --- | --- |
| **Symbol** | **Entrez Gene Name** | **Expected** | **Location** | **Types** |
| ABL2 | ABL proto-oncogen2 | - | C | TR |
| ARHGAP9 | Rho GTPase activating protein | ⇓Down | C | TR |
| CDC42EP1 | CDC42 effector protein | ⇑Up | ES | E |
| CIT | Citron rho-interacting | ⇑Up | C | other |
| NGEF | Neuronal guanine nucleotide | ⇑Up | C | other |
| TTN | Titin |  | C | other |

| **C.** |  |  |  |  |
| --- | --- | --- | --- | --- |
| **Symbol** | **Entrez Gene Name** | **Location** | **Types** | **Biomarker application** |
| FZD6 | Frizzled class receptor 6 | PM | GPCR | - |
| HDAC1 | Histone deacetylase 1 | N | TR | Efficacy |
| HDAC2 | Histone deacetylase 2 | N | TR | - |
| LPIN1 | Lipin 1 |  | P |  |
| SENP2 | SUMO/sentrin/SMT3 specific | N | PP | - |

| **D.** |  |  |  |  |  |
| --- | --- | --- | --- | --- | --- |
| **Symbol** | **Entrez Gene Name** | **Expected** | **Location** | **Types** | **Biomarker application** |
| HDAC1 | Histone deacetylase 1 | - | N | TR | Efficacy |
| HDAC2 | Histone deacetylase 2 | - | N | TR | - |
| TEP1 | Telomerase-associated protein 1 | ⇑Up | N | E | - |
| TERF2 | Telomeric repeat binding factor 2 | - | N | other | Diagnosis |

| **E.** |  |  |  |  |
| --- | --- | --- | --- | --- |
| **Symbol** | **Entrez Gene Name** | **Location** | **Types** | **Biomarker application** |
| HDAC1 | Histone deacetylase 1 | N | TR | Efficacy |
| HDAC2 | Histone deacetylase 2 | N | TR | - |
