# Supplementary material for: A systems medicine approach for finding target proteins affecting treatment outcomes in patients with non-Hodgkin lymphoma
Source: PLoS One. 2017 Sep 11;12(9):e0183969. doi: 10.1371/journal.pone.0183969 (PMC5593188; doi:10.1371/journal.pone.0183969)
Supplement: S6 Table — (A) Neuropathic pain signaling in dorsal horn neurons (Ratio: 7/100 (0.07) and P-value: 2.07E-05) and synaptic long term potentiation (Ratio: 7/119 (0.059) and P-value: 6.34E-05), (B) CREB signaling in neurons (Ratio: 8/171 (0.047) and P-value: 9.63E-05), (C) Dopamine-DARPP32 feedback in cAMP signaling (Ratio: 7/161 (0.043) and P-value: 4.12E-04), and (D) nNOS signaling in neurons (Ratio: 4/47 (0.085), P-value: 6.30E-04). nNOS: Neuronal nitric oxide synthase, TR: Transcription regulator, C: Cytoplasm, E: Enzyme, PM: Plasma membrane, K: Kinase, GPCR: G-protein coupled receptor, and IC: Ion channel. (DOCX) [file pone.0183969.s009.docx]

**S6 Table. Top canonical pathways at PS gene signature identified through IPA.** (A) Neuropathic pain signaling in dorsal horn neurons (Ratio: 7/100 (0.07) and *P*-value: 2.07E-05) and synaptic long term potentiation (Ratio: 7/119 (0.059) and *P*-value: 6.34E-05), (B) CREB signaling in neurons (Ratio: 8/171 (0.047) and *P*-value: 9.63E-05), (C) Dopamine-DARPP32 feedback in cAMP signaling (Ratio: 7/161 (0.043) and *P*-value: 4.12E-04), and (D) nNOS signaling in neurons (Ratio: 4/47 (0.085), *P*-value: 6.30E-04). nNOS: Neuronal nitric oxide synthase, TR: Transcription regulator, C: Cytoplasm, E: Enzyme, PM: Plasma membrane, K: Kinase, GPCR: G-protein coupled receptor, and IC: Ion channel.

| **A.** |  |  |  |  |  |
| --- | --- | --- | --- | --- | --- |
| **Symbol** | **Entrez Gene Name** | **Expected** | **Location** | **Types** | **Biomarker application** |
| CAMK2A | Calcium/calmodulin-dependent kinase 2 alpha | ⇑Up | C | K | - |
| GRIN2B | Glutamates receptor, ionotropic, N-methyl D-aspartate 2B | ⇑Up | PM | IC | diagnosis |
| GRIN2D | Glutamates receptor, ionotropic, N-methyl D-aspartate 2D | ⇑Up | PM |  | - |
| GRM5 | Glutamate receptor, metabotropic 5 | ⇑Up | PM | GPCR | - |
| PLCG1 | Phospholipase C, gamma 1 | ⇑Up | C | E | - |
| PLCG2 | Phospholipase C, gamma 2 | ⇑Up | C | E | - |
| PRKD1 | Protein kinase D1 | ⇑Up | C | E | - |

| **B.** |  |  |  |  |  |
| --- | --- | --- | --- | --- | --- |
| **Symbol** | **Entrez Gene Name** | **Expected** | **Location** | **Types** | **Biomarker application** |
| CAMK2A | Calcium/calmodulin-dependent kinase 2A | ⇑Up | C | K | - |
| GRID1 | Glutamates receptor, ionotropic, Delta 1 | ⇑Up | PM | IC |  |
| GRIN2B | Glutamates receptor, ionotropic, N-methyl D-aspartate 2B | ⇑Up | PM | IC | diagnosis |
| GRIN2D | Glutamates receptor, ionotropic, N-methyl D-aspartate 2D | ⇑Up | PM | IC | - |
| GRM5 | Glutamate receptor, metabotropic 5 | ⇑Up | PM | GPCR | - |
| PLCG1 | Phospholipase C, gamma 1 | ⇑Up | C | E | - |
| PLCG2 | Phospholipase C, gamma 2 | ⇑Up | C | E | - |
| PRKD1 | Protein kinase D1 | ⇑Up | C | E | - |

| **C.** |  |  |  |  |  |
| --- | --- | --- | --- | --- | --- |
| **Symbol** | **Entrez Gene Name** | **Expected** | **Location** | **Types** | **Biomarker application** |
| CACNA1A | Calcium channel, voltage- dependent, P/Q type, alpha 1A subunit | ⇑Up | PM | IC | - |
| CACNA1S | Calcium channel, voltage- dependent, L type, alpha 1S subunit | ⇑Up | PM | IC | - |
| GRIN2B | Glutamates receptor, ionotropic | - | PM | IC | diagnosis |
| GRIN2D | Glutamates receptor, ionotropic | - | PM | IC | - |
| PLCG1 | Phospholipase C, gamma 1 | ⇑Up | C | E | - |
| PLCG2 | Phospholipase C, gamma 2 | ⇑Up | C | E | - |
| PRKD1 | Protein kinase D1 | ⇑Up | C | E | - |

| **D.** |  |  |  |  |  |
| --- | --- | --- | --- | --- | --- |
| **Symbol** | **Entrez Gene Name** | **Expected** | **Location** | **Types** | **Biomarker application** |
| CAMK2A | Calcium/calmodulin-dependent kinase 2A | ⇑Up | C | K | - |
| GRIN2B | Glutamates receptor, ionotropic, N-methyl D-aspartate 2B | ⇑Up | PM | IC | diagnosis |
| GRIN2D | Glutamates receptor, ionotropic, N-methyl D-aspartate 2D | ⇑Up | PM | IC | - |
| PKD1 | Protein kinase D1 | - | C | K | - |
